# Buruli Ulcer in Cameroon: The Development and Impact of the National Control Programme

**DOI:** 10.1371/journal.pntd.0004224

**Published:** 2016-01-13

**Authors:** Earnest Njih Tabah, Dickson Shey Nsagha, Anne-Cécile Zoung-Kanyi Bissek, Alfred Kongnyu Njamnshi, Martin W. Bratschi, Gerd Pluschke, Alphonse Um Boock

**Affiliations:** 1 National Yaws, Leishmaniasis, Leprosy and Buruli ulcer Control Programme, Ministry of Public Health, Yaounde, Cameroon; 2 Swiss Tropical and Public Health Institute, Basel, University of Basel, Basel, Switzerland; 3 Faculty of Medicine and Biomedical Sciences, The University of Yaounde 1, Yaounde, Cameroon; 4 Department of Public Health and Hygiene, Faculty of Health Sciences, University of Buea, Buea, Cameroon; 5 Department of Operational Research in Health, Ministry of Public Health, Yaounde, Cameroon; 6 Department of Neurology, Central Hospital, Yaounde, Cameroon; 7 Regional Bureau for Africa of the FAIRMED Foundation, Yaounde, Cameroon; University of Tennessee, UNITED STATES

## Abstract

**Background:**

Cameroon is endemic for Buruli ulcer (BU) and organised institutional BU control began in 2002. The objective was to describe the evolution, achievements and challenges of the national BU control programme (NBUCP) and to make suggestions for scaling up the programme.

**Methods:**

We analysed collated data on BU from 2001 to 2014 and reviewed activity reports NBUCP in Cameroon. Case-detection rates and key BU control indicators were calculated and plotted on a time scale to determine trends in performance. A linear regression analysis of BU detection rate from 2005–2014 was done. The regression coefficient was tested statistically for the significance in variation of BU detection rate.

**Principal findings:**

In 14 years of BU control, 3700 cases were notified. The BU detection rate dropped significantly from 3.89 to 1.45 per 100 000 inhabitants. The number of BU endemic health districts rose from two to 64. Five BU diagnostic and treatment centres are functional and two more are planned for 2015. The health system has been strengthened and BU research and education has gained more interest in Cameroon.

**Conclusion/Significance:**

Although institutional BU control Cameroon only began 30 years after the first cases were reported in 1969, a number of milestones have been attained. These would serve as stepping stones for charting the way forward and improving upon control activities in the country if the major challenge of resource allocation is dealt with.

## Author Summary

Organised control of Buruli ulcer (BU), the third most common mycobacterial disease, began in Cameroon in 2002. We used 2001–2014 data and activity reports from the National BU Control Programme, to describe the onset, evolution, achievements and challenges of BU control in Cameroon, and make suggestions for improvement and expansion of programme activities. A cumulative number of 3700 BU cases have been detected and treated free-of-charge in five functional BU diagnostic and treatment centres. Sixty-four BU-endemic districts are now confirmed in Cameroon. Furthermore, Cameroon has actively participated in several BU research activities. However major challenges regarding demanding and costly surveillance, education, diagnostic and treatment activities remain to be addressed. Capitalizing on experiences and lessons learned in the past 14 years of activities, the National BU control programme should however be prepared to move forward.

## Introduction

Buruli ulcer (BU), caused by *Mycobacterium ulcerans*, is the third most common mycobacterial infection, after tuberculosis and leprosy. *M*. *ulcerans* infection leads to chronic necrotizing ulcers [1], resulting in deformities, functional limitation and social stigma, if left untreated [2; 3].

BU occurs in tropical and sub-tropical regions near stagnant or slow flowing water bodies and marshlands [4; 5], and worldwide, this association has been shown to be a risk factor for the infection. The mode of transmission however is unclear, although some studies have suggested the involvement of an animal reservoir or of insect vectors [6].

BU affects people of all ages and both sexes, but mostly children below 15 years of age. However, children below five years seem to be underrepresented among patients [7] and less exposed to *M*. *ulcerans* than older children [8]. Although all body parts may be involved, about 90% of lesions occur on the limbs [7], which may reflect the mode of transmission. Since *M*. *ulcerans* is thermosensitive, it is primarily causing skin lesions.

**Fig 1:**
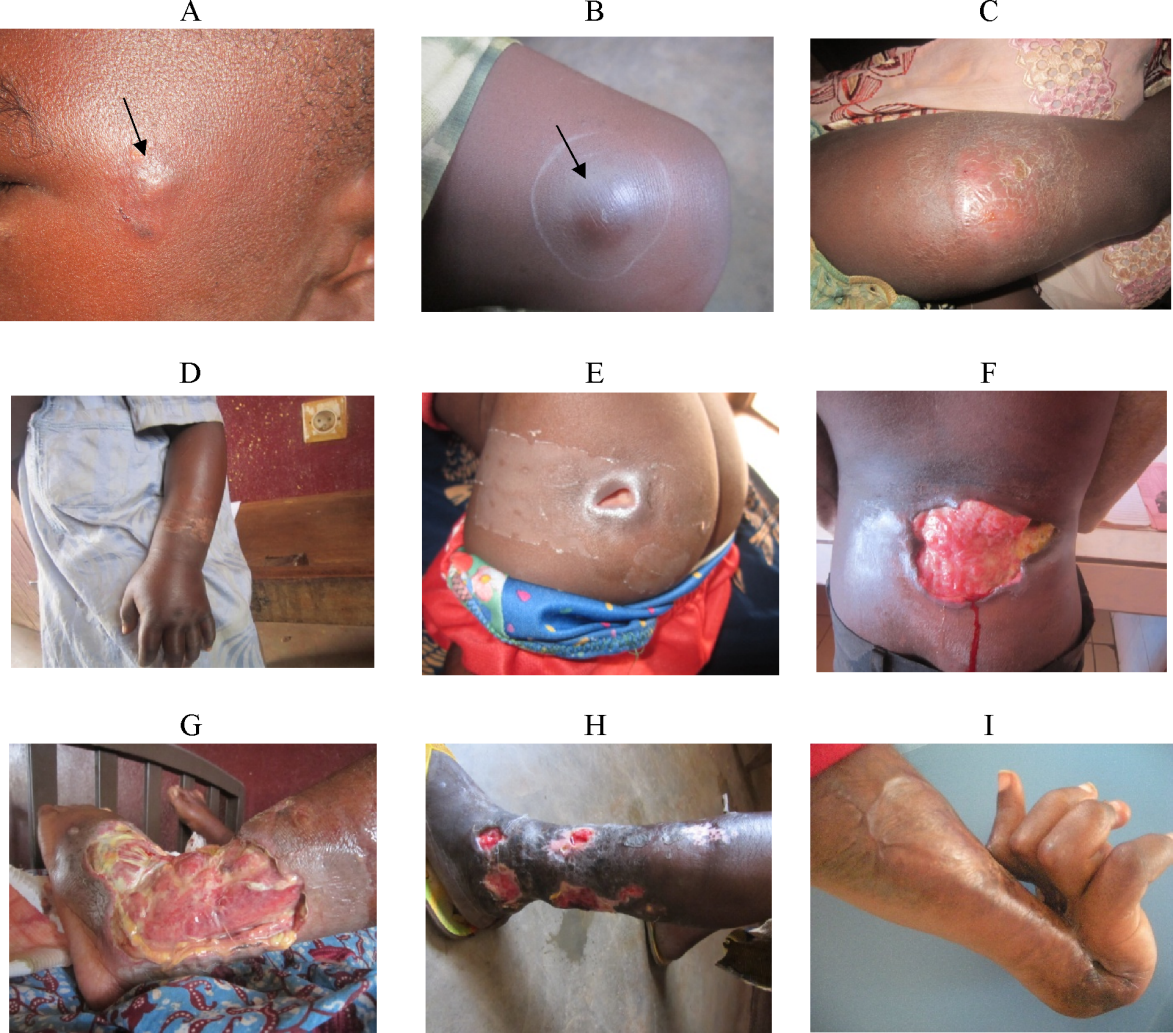
Clinical manifestations of BU cases seen in Cameroon. A. Papule: a swollen and non painful cutanous lesion of 003C1cm in diameter; B. Nodule: a swollen and non painful lesion extending from the skin to the sub-cutanous tissue, with a diameter of 1–2cm; C. Plaque: a firm and non painful induration of the skin with well delimited borders, usually with desqamation of the affected skin surface; D. Oedema: a diffuse and extended and non pitting swelling which is firm, non painful and has no clearly defined borders; E. Category I ulcer: a small ulcerated and mildly painful lesion of <5cm of diameter with undermined borders; F. Category II ulcer: an ulcerated lesion with undermined borders and a diameter of 5–15cm; G. Category III ulcer: a large ulcerated lesion of >15cm of diameter with indurated undermined borders, commonly has necrotic tissue on the ulcer bed; H. Disseminated BU lesions; I. Sequelae of BU: a viscious healing of a poorly treated BU lesion with complete retraction of the hand in an extesion position.

BU presents in two major clinical forms: non-ulcerative (papule, nodule, plaque, and oedema) and ulcerative lesions (Fig 1). The WHO has defined three categories of lesions based on their sizes and location on the body [9]. BU can be diagnosed clinically by experienced and skilled health workers in endemic areas [10]. Four standard laboratory methods can be used to confirm BU [11], however, polymerase chain reaction (PCR) targeting the multi-copy insertion segment (IS) 2404 of *M*. *ulcerans* is the most commonly used because it has the highest sensitivity and results can be available within 48 hours [12]. The treatment of BU has evolved from exclusively surgical [13] to the use of specific antibiotics [10; 14] in association with, simple surgery for controlled wound healing, physiotherapy, nutritional and psychosocial support if required. Today, major surgery is reserved only for treatment of BU complications.

Effective BU control began in Cameroon in 2002. In this article, we describe the national BU control programme (NBUCP) in Cameroon from its creation to 2014, based on a review of reports and data from 2001 to 2014 compiled at the NBUCP. The objective is to describe the evolution, achievements, and challenges of the NBUCP, and make suggestions for improvement and expansion of BU programme activities. The paper shall be useful for advocacy to mobilize resources for implementation of BU control programme activities worldwide.

## Methods

We analysed collated data on BU from 2001 to 2014 and reviewed activity reports available NBUCP office. In Cameroon, BU is diagnosed and treated at BU diagnostic and treatment centres (BU-DTCs). Within the framework of national BU surveillance, all BU-DTCs are provided with BU case-definition, diagnostic and treatment guidelines and other documentation on the disease for use by the staff. Standard WHO patient case record files and registers are used at BU-DTCs to document information on each BU patient. The data recording and reporting process at BU-DTCs is regularly monitored and supervised by the NBUCP to ensure correctness and completeness. The data is compiled and transmitted together with activity reports monthly to the NBUCP where it is captured electronically.

The data on BU cases was classified by year and health district of origin. The health districts that registered at least a case of BU between 2001 and 2014 were considered as districts at risk of BU and their annual populations for 2005 were obtained from the report of the 3rd general population and housing census of Cameroon [15]. The populations of these districts for the periods 2001–2004 and 2006–2014 were estimated on the bases of a population growth rate of 2.8% and 2.6% respectively [15]. Aggregates of the district populations were used to calculate annual BU detection rates per 100 000 inhabitants. The annual number of BU cases, the cumulative number of cases and the annual detection rates were plotted on a time scale to determine trends. Key BU control performance indicators [16] were calculated as proportions and plotted on a time scale to determine trends in the performance of the NBUCP. The performance indicators considered were the proportions of ulcerative lesions, category 3 lesions, cases confirmed by PCR, cases below 15 years of age and lesions located on limbs. A linear regression analysis of BU detection rate from 2005–2014 was done. The regression coefficient was tested statistically for the significance in variation of BU detection rate. Shape files of the Cameroon health district map obtained from the Sub-department for epidemiological surveillance in the Ministry of Public Health was used to produce the various BU maps of Cameroon, using the ArcGIS version 9 mapping software, to further illustrate trends.

### Ethics statement

This study was instructed by the Cameroon Ministry of Public Health Decision No 0486/D/MINSANTE/CAB and was approved the National Ethics Committee of Cameroon through the authorisation No 041/CNE/DNM/09. All data were anonymized and confidentiality was strictly respected in the data handling and analysis.

## Results and Discussion

### BU control in Cameroon: The creation and the evolution of the NBUCP

Although BU was first reported in Cameroon in 1969 [17], control activities only began effectively 33 years later, in 2002. The triggers for control activities in Cameroon were the new momentum to BU control following the 1998 Yamoussoukro Declaration [18] and the reconfirmation of the Nyong Basin in Central Cameroon as a BU endemic area by Noeske and colleagues in 2001, when they identified 436 clinical cases of BU, 162 of whom were sampled and 135 confirmed by IS2404 PCR [19]. Noeske’s findings led to the creation of the first two BU-DTCs in the country in 2002, in Ayos and Akonolinga both of which are located within the Nyong basin. These BU-DTCs were set-up with the support of two NGOs: FAIRMED (former Aide aux Lépreux Emmaüs Suisse) and Médecins Sans Frontières Suisse (MSF-CH), respectively. The effectiveness of the treatment of BU in these pioneer centres quickly led Cameroon’s Ministry of Public Health to create the NBUCP in 2004, underlying the important role of research in health development policy.

The initial goals and objectives of the NBUCP largely reflected those of the WHO GBUI as stated in the Yamoussoukro Declaration” [18]. The NBUCP objectives have however evolved over time, to suit new developments and orientations in the domain as charted by the 2004 WHA resolution on BU and the 2009 Cotonou Declaration on BU.

The current goal of the NBUCP is to reduce the suffering of the population due to BU. The global objective is to ensure accessibility to quality health services in endemic areas in order to reduce morbidity and disability linked to BU in Cameroon. Specifically, the NBUCP aims to detect cases of BU early, preferentially as non-ulcerative forms and category 1 lesions; to confirm 70% of clinical BU cases by PCR; to treat all active cases of BU according to standard guidelines; and to heal at least 95% of BU patients without limitation of joint movement.

### Major achievements of BU control in Cameroon

#### Health system strengthening through case finding, management, and public-private partnerships

The pivot of BU control in Cameroon has been the BU-DTCs situated in major endemic foci. The BU-DTC facilities were built or rehabilitated and equipped by support partners to provide adequate infrastructure for BU diagnosis and treatment, including surgical theatres, wound dressing rooms, laboratories, physiotherapy units and admission wards. Health staff of the BU-DTCs received on-the-job training through workshops facilitated by experts, using the WHO training modules and guidelines on BU care. These training sessions included aspects such as wound management, which are also useful for general patient care. Health staff also received regular refresher training and supportive supervision from the NBUCP and partner NGOs.

Initially case-finding was active, with medical teams from BU-DTCs carrying out mass screening for BU, sensitization and awareness campaigns in communities and schools. Gradually, health committees were activated in the intervention areas, and they became responsible for community sensitization and awareness campaigns about BU. Community participation increased as many volunteers were trained to recognize BU in their communities and refer suspected cases to the BU-DTC for confirmation and treatment. As communities became more aware of BU, the active case-finding gradually gave way to passive case-finding with cases coming on their own or being referred to BU-DTCs.

#### BU case detection and management

**Fig 2:**
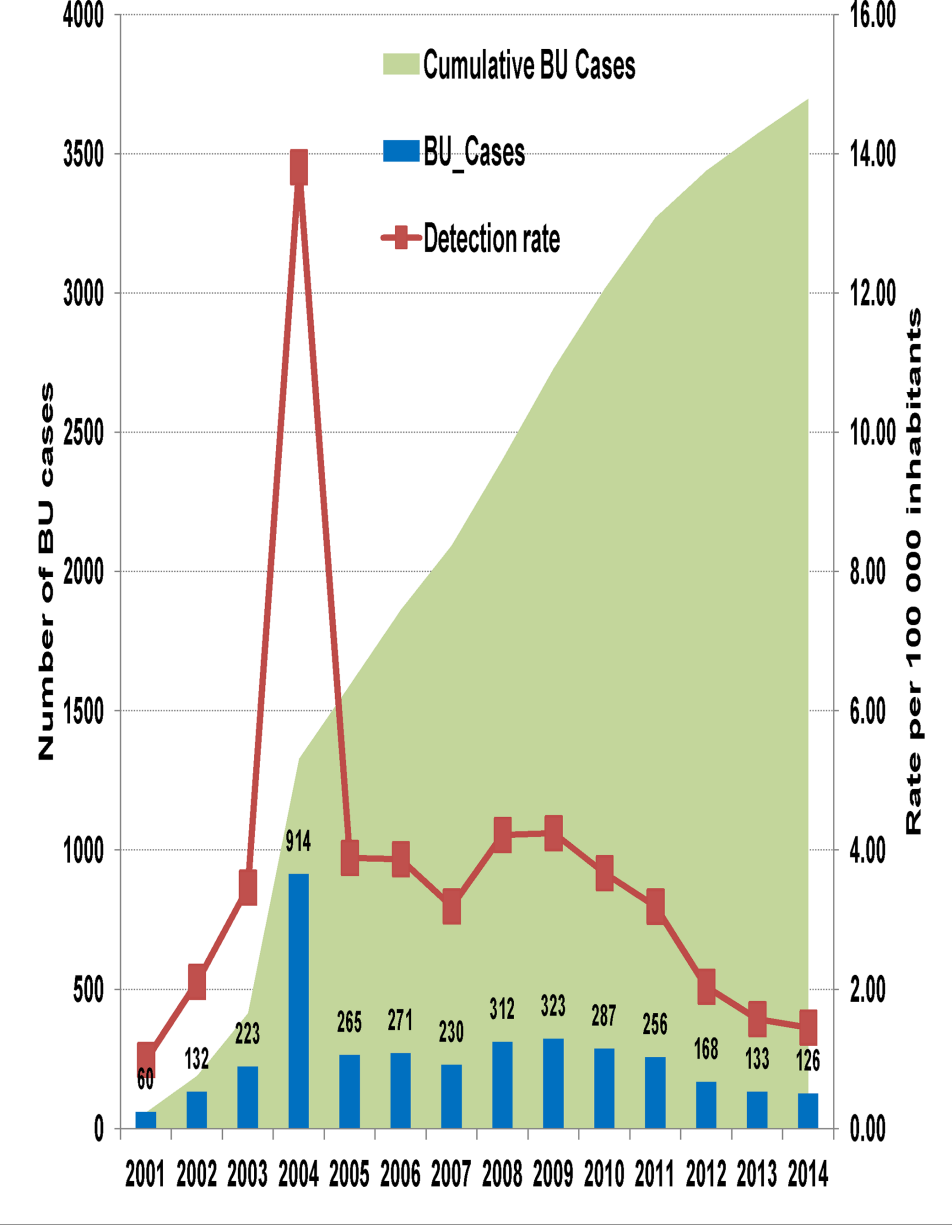
Trend in BU case notification between 2001 and 2014 in Cameroon. A cumulative number of 3700 BU cases were notified between 2001 and 2014, with an annual average case notification of 264 cases. The peak in 2004 is attributed to the national BU survey in that year. There is a progressive reduction in case notification since 2005. The annual BU detection rate increased from 0.99 in 2001 to 3.89 per 100 000 inhabitants in 2005 and dropped progressively to reach 1.45 per 100 000 inhabitants cases in 2014.

Fig 2 shows the trend in BU case notification between 2001 and 2014 at BU-DTCs in Cameroon. A cumulative total of 3700 clinically confirmed BU patients have been diagnosed and treated. The annual average case detection was 264 over the 14-year period. The peak in 2004 is attributed to the national survey in that year [20]. This peak may imply that a proportion of cases remain unnoticed by the public health system and that the real annual case detection rate of BU in Cameroon is much higher than the annual average of 3.69 per 100 000 inhabitants over this 14-year period.

**Fig 3:**
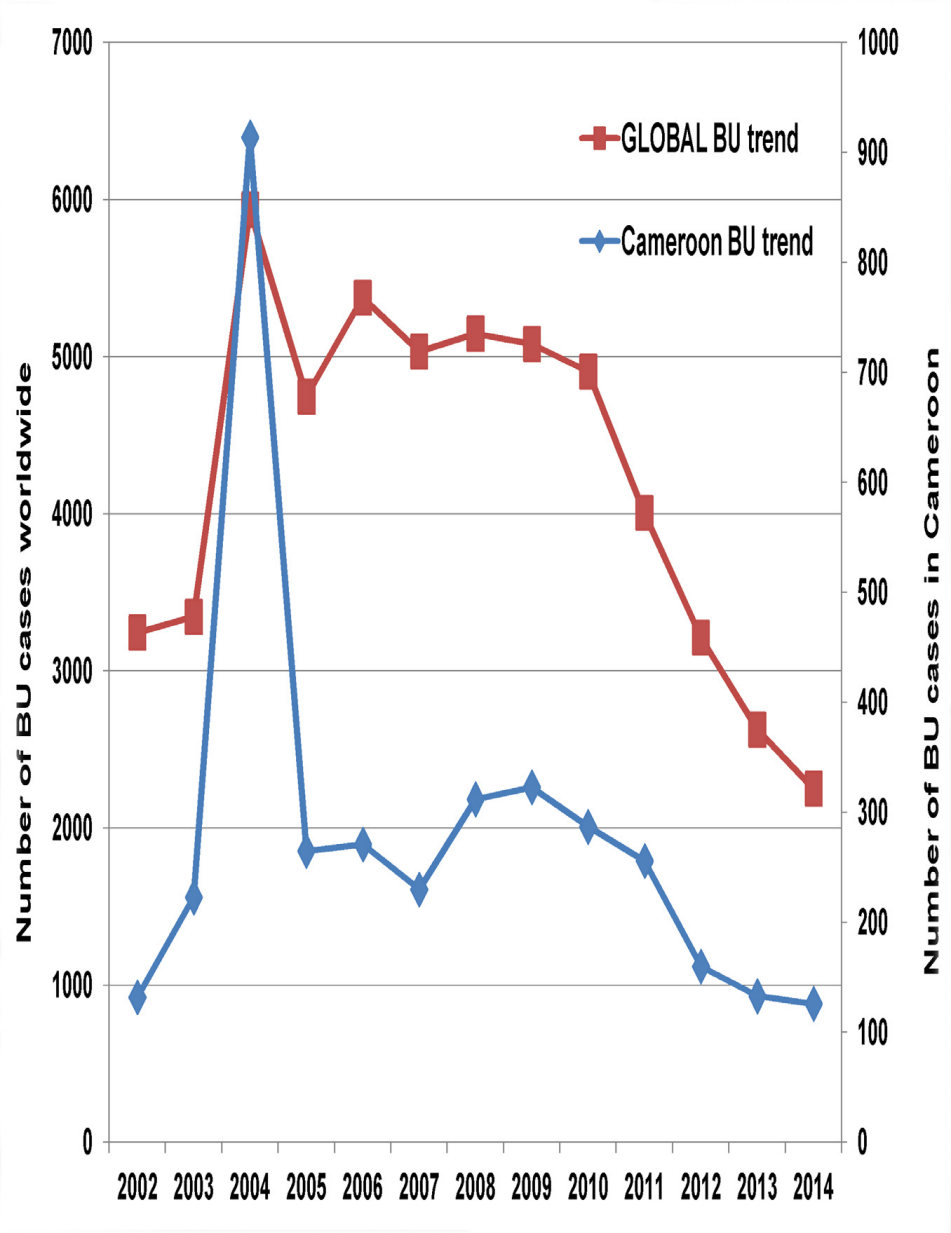
Comparison between the Cameroon national and the global trend in BU cases from 2002 to 2014. The trend in the global BU cases was similar to that of Cameroon with a rise from 3245 cases in 2002 to a peak at 5937 in 2004, followed by a progressive reduction to reach 2250 in 2014.The global BU data was downloaded from the WHO website at http://apps.who.int/gho/indicatorregistry/App_Main/view_indicator.aspx?iid=2448

The annual BU detection rate increased from 0.99 in 2001 to 3.89 per 100 000 inhabitants in 2005, and then dropped progressively to reach 1.45 per 100 000 inhabitants cases in 2014. The reduction was significant (regression coefficient: -0.29 (95%CI: -0.45 to -0.12), P = 0.004). This decreasing trend is comparable to the worldwide trend, with a 52.6% reduction in BU cases between 2005 and 2014 [21] (Fig 3).

The exact reasons for the reduction in BU cases in the world are not known [12]. Although in Cameroon active BU case finding in the Nyong basin has greatly decreased, we cannot attribute the reduction in BU cases only to this, given that in the Bankim endemic area where activities are sustained; there has been a 46% reduction in BU cases between 2008 and 2014. Could it be that the continuous case finding and treatment of BU for over one decade has led to the reduction in its incidence, or does BU has a cyclical pattern of occurrence and that we are coming to the end of a cycle? We suggest that, under the auspices of the WHO, the scientific community should carry out a multicentre study to determine the reasons for the reduction of BU cases worldwide.

**Fig 4:**
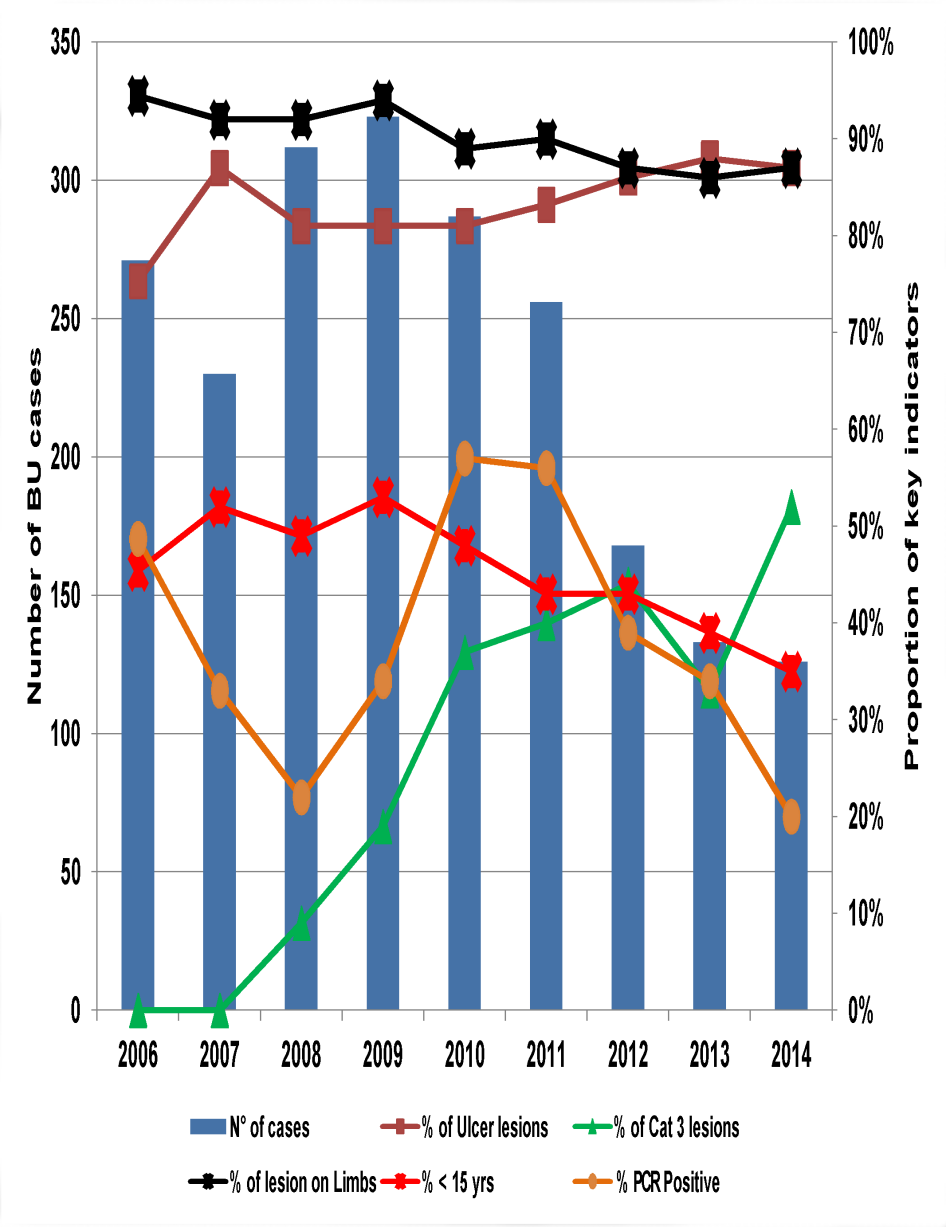
Trends in key BU control indicators from 2006 to 2014. Notice that during this period, the ulcerative form of BU constituted about 83% of lesions and 90% of all lesions were located on the limbs. On average, children below 15 years of age constituted 45% of cases. The proportion of category 3 lesions rose from 9% in 2008 to 52% in 2014 and the proportion of BU cases confirmed by PCR dropped from 57% in 2010 to 20% in 2014.

A nine-year trend in key BU control indicators (Fig 4) reveals that the ulcerative form of BU constituted about 83% of lesions and 90% of all lesions were located on the limbs. On average, children below 15 years of age constituted 45% of cases. The proportion of category III lesions rose from 9% in 2008 to 52% in 2014, two-fold the new WHO target of ≤ 25% [22]. The proportion of BU cases confirmed by PCR dropped from 57% in 2010 to 20% in 2014, far below the new WHO target of 70% [22].

The NBUCP has depended largely on external partner funding from inception in 2002 for the implementation of its activities. This partner funds have dwindled rapidly since 2010. The consequences have been the decrease in BU surveillance and confirmation of cases by PCR, explaining the progressive rise in Category III lesions and drop in the proportion of cases confirmed by PCR. To remedy this situation, the NBUCP has embarked on an aggressive advocacy with hierarchy of the Ministry of Health and non-governmental organisations (NGOs) to source additional funding for BU activities in general and the improvement of BU surveillance and PCR confirmation in particular. The Ministry of Health has begun to respond by instituting a budgetary line for BU activities since 2014, and one NGO has opted to pay for all confirmation by PCR in 2015. The challenge of the NBUCP shall be to sustain these offers.

#### Expansion of BU control programme coverage

**Fig 5:**
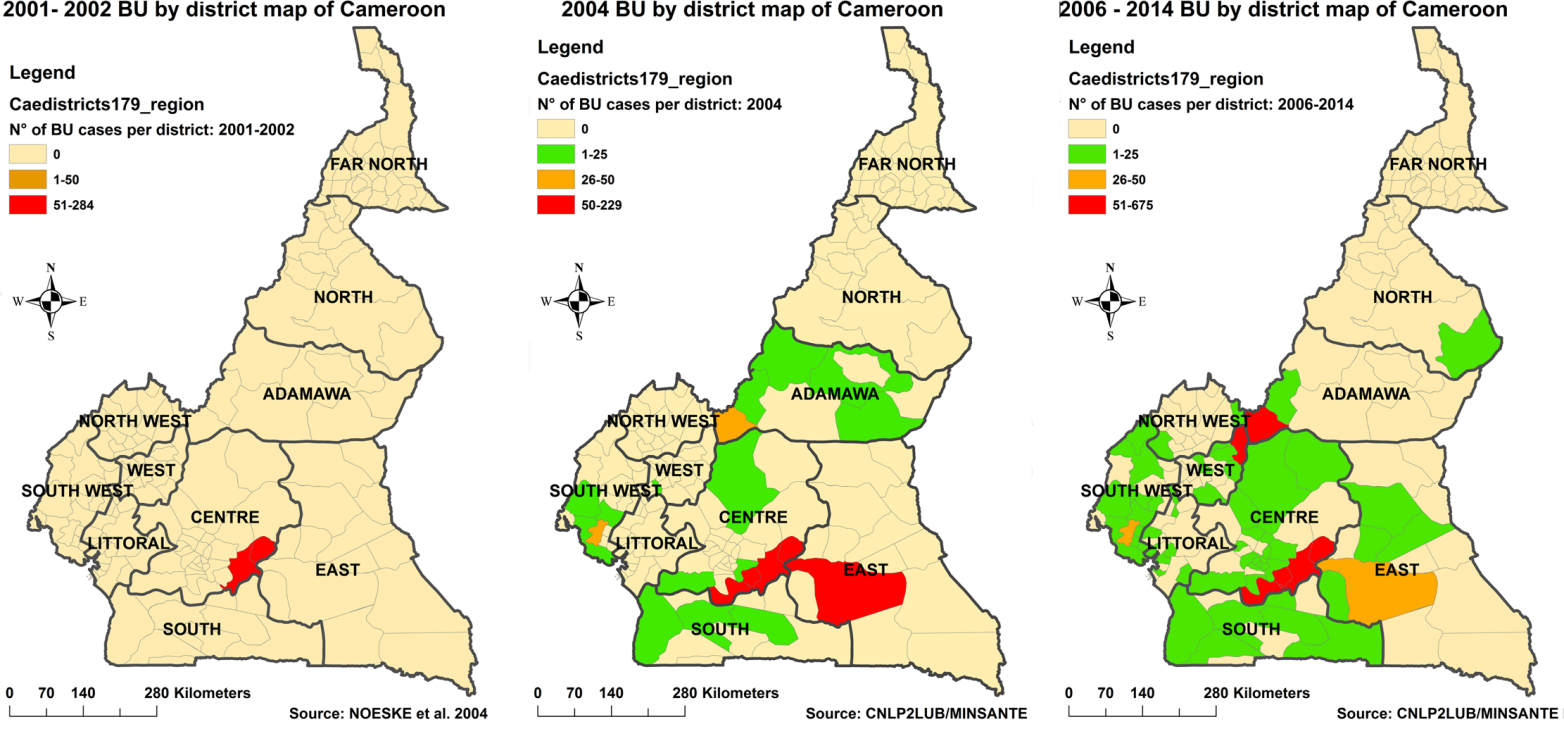
Evolution of confirmed BU endemic health districts between 2001 and 2014. During the period 2001–2002 (A), the only known BU endemic health districts were Ayos and Akonolinga in the Nyong basin of central Cameroon [19]. The first 2 BU-DTCs were created in these districts in 2002 to begin BU care in Cameroon. The national BU survey in 2004 following the creation of the NBUCP revealed the presence of BU in 19 other health districts (B). Three new BU-DTCs were created in 2006 in the 3 most endemic of the 19 health districts namely Bankim in the Adamawa region, Mbonge in the South west Region and Ngoantet-Mbalmayo in the Centre Region. From 2006–2014, the 5 BU-DTCs in the country have treated BU cases originating from sixty-four health districts (C).

Upon creation in 2004, the NBUCP developed its first national BU control strategic plan. Within the framework of the plan, the first national BU survey conducted in 2004 revealed the presence of BU in 19 health districts [20] in addition to the Ayos and Akonolinga Districts in the Nyong basin (Fig 5A & 5B). In 2006, programme activities were extended, through the creation of BU-DTCs in three most endemic of the newly identified health districts namely: Bankim, Ngoantet-Mbalmayo and Mbonge, bringing to five the total number of BU-DTCs nationwide. They are located in the Adamaoua, South-West and Centre regions of the country respectively.

A study of origins of BU cases notified at the five BU-DTCs from 2006 to 2014 revealed that cases came from sixty-four health districts around the country (Fig 5C). The endemic health districts confirmed between 2000 and 2004 have remained the hot spots of BU all along. However neighbouring health districts have consistently notified BU during this period. The need for further expansion of BU activities to these new foci is clear. The NBUCP plans to create two more BU-DTCs in 2015. Furthermore there is need for another national survey on BU. Such a survey may confirm the presence of BU in the northern part of the country, given that in 2008 a case from a district in the North region was confirmed and treated at the Akonolinga BU-DTC.

#### Research accompanying BU control in Cameroon

Cameroon has participated in research on BU since the launching of the GBUI in 1998. Indigenous research projects have been carried out in the country and Cameroonian researchers have participated in research consortia organized by international research institutes. Results from these research ventures have contributed to shape the knowledge pool available on BU today and has influenced health policy in the country.

BU transmission studies in Cameroon have identified water bug families that harbour *M*. *ulcerans* in their salivary glands [23; 24]. Three of them are good flyers, are attracted by light sources, and do bite humans. The authors suggested that these bugs could play a role in disseminating *M*. *ulcerans* as hosts and vectors [24]. There is however no consensus on this within the scientific community [25].

Persistence of *M*. *ulcerans* specific DNA sequences over a period of more than two years has been observed at a water contact location of BU patients in a village of the Bankim endemic area, after successful treatment of all local patients [26]. At defined positions in a shallow water hole used by the villagers for washing and bathing, detritus remained consistently positive for *M*. *ulcerans* DNA. Underwater decaying organic matter may thus represent a reservoir of *M*. *ulcerans* for direct infection of skin lesions or vector-associated transmission.

A seroepidemiological study, also carried out in the Bankim area demonstrated that sera collected from children below the age of four do not contain antibodies against the 18KDa small heat shock protein of *M*. *ulcerans* [8]. These data suggest that exposure to *M*. *ulcerans* increases at an age which coincides with the children moving further away from their homes and having more intense environmental contact, including exposure to water bodies at the periphery of their villages [7].

A study in Ayos and Akonolinga [27] revealed that in a context of free medical treatment of BU, the cost burden constituted 25% of the annual household earnings of affected families, far above the acceptable 10% recommended by the WHO for health expenditure. Further studies on innovative and more cost-effective interventions with greater involvement of the communities are required in order to deal with the high cost burden on affected families.

The Ayos BU-DTC hosted the thermotherapy trial as an alternative treatment for BU [28]. This proof-of-principle trial demonstrated that heat application alone was effective in treating six category I BU lesions with no relapses after 1½ years of follow-up. The authors suggested that heat application device used could be suitable to treat BU in resource-poor settings like in Cameroon.

#### Milestones in BU control in Cameroon

**Fig 6:**
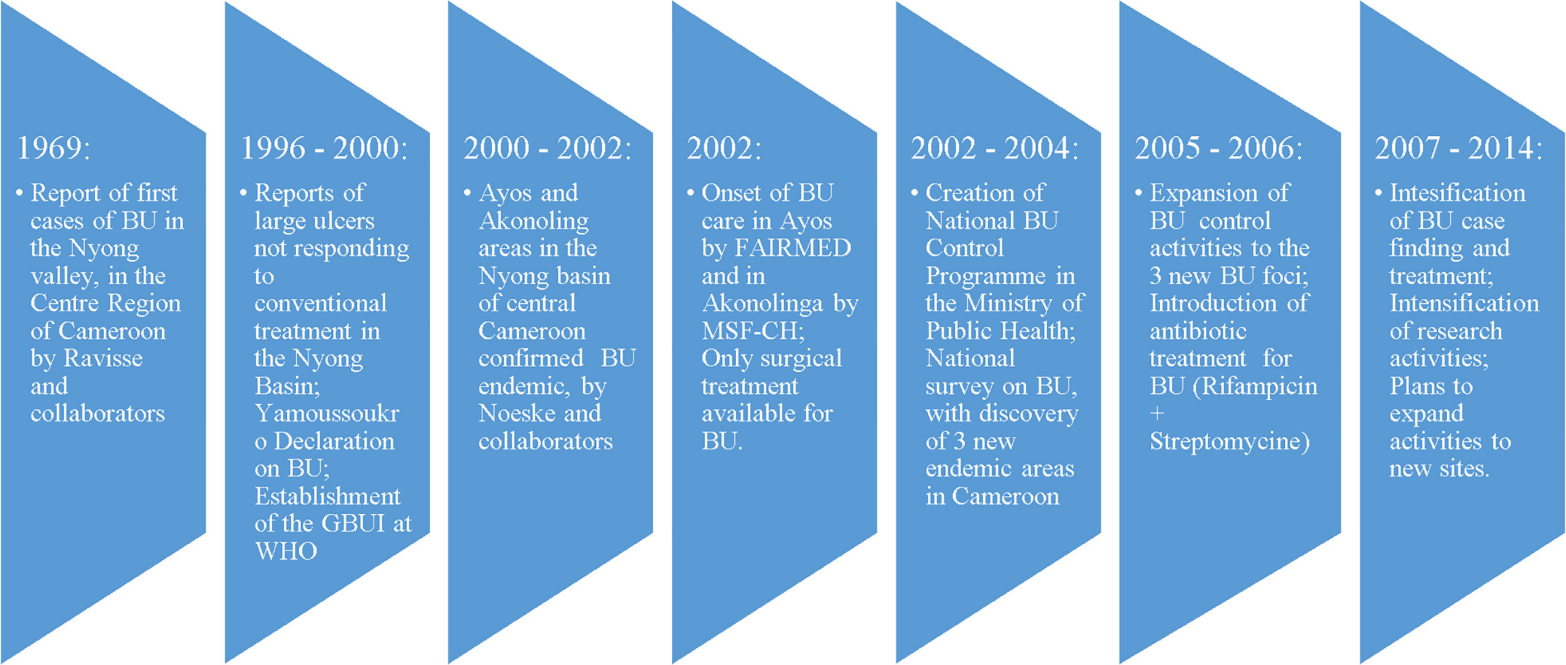
Milestones in the evolution of BU control activities in Cameroon, 1969–2014. Although institutional BU control Cameroon only began 30 years after the first cases were reported in 1969, a number of milestones have been attained. These will serve as stepping stones for charting the way forward and improving upon control activities in the country.

A number of important milestones have been attained in fourteen years of BU control in Cameroon (Fig 6).

### Challenges in BU control

BU is an expensive condition to treat and occurs primarily in poor and remote parts of Cameroon. Partner resources, upon which NBUCP activities largely depended, are becoming rarer leading to cutting down on activities and consequent drop in performance indicators. The Government of Cameroon funding for recurrent costs of programme activities is very limited and cannot cover the gap. This situation does not allow for rapid expansion of BU activities to new endemic districts.

There is a lack of diagnostic and management skills for BU among the health personnel. BU care is not taught in all faculties of medicine and training schools for health personnel. Consequently, health personnel may only learn about BU on the job, if they get posted to a BU-DTC, through workshops and refresher courses organised by the NBUCP and partners. A series of workshops on the management of chronic wounds and BU are being organised since 2013 in collaboration with MSF-CH, Hôpitaux Universitaires de Genève and the Faculty of Medicine and Biomedical Sciences of the University of Yaounde 1 (FMBS-UY1).

Many unanswered questions about BU still prevail. The mode of transmission, the exact prevalence and incidence, the relationship between BU and HIV/AIDS, the role of beliefs and customs surrounding BU, economic costs of BU treatment and the effect of *M*. *ulcerans* infection on nerve conduction are a few of the concerns. These questions call for well planned and executed research projects. Most research projects in Cameroon so far were championed by expatriates. However, this trend is changing as more Cameroonian researchers are now involved, and the FMBS-UY1 has now introduced a course on BU in its curriculum. Furthermore, some Cameroonian researchers and care providers have recently founded the Cameroon Wound Management Society with the aim, among others, of promoting BU research.

### The way forward

Much experience has been acquired in 12 years of BU control in Cameroon. Capitalizing on these experiences and lessons learned should prepare the NBUCP to be more effective and efficient. This will require dealing with the identified challenges. The NBUCP is intensifying advocacy to get more partners on board as well as secure more resources from traditional partners of the programme. The Cameroon’s Ministry of Public Health is aware of the dwindling partner resources and has begun to put in some additional resources for recurrent costs since 2013.

The remaining faculties of medicines of the various state universities as well as the training schools for health personnel in Cameroon should follow the example of FMBS-UY1 to include courses on BU in their training programmes and be more involved in research activities on the disease.

### Conclusion

This review shows that significant progress has been made in BU case detection and management, health system reinforcement, capacity building and research in Cameroon, although institutional BU control Cameroon only began 30 years after the first cases were reported. However the reduction in programme resources and activities by the major support partners has led to a steady drop in performance indicators since 2010. More effort needs to be made in resource mobilization for expansion and scaling up of the programme activities.

## Supporting Information

S1 Dataset For Figs 2 and 3: Trends in BU case notification between 2001 and 2014 in Cameroon and globally. In Cameroon, a cumulative number of 3700 BU cases were notified between 2001 and 2014, with an annual average case notification of 264 cases. The peak in 2004 is attributed to the national BU survey in that year. There is a general drop in case notification since 2010. The trend in the global BU cases was similar to that of Cameroon with a rise from 3245 cases in 2002 to a peak at 5937 in 2004, followed by a progressive reduction to reach 2250 in 2014 [21].
(XLSX)

S2 Dataset For Fig 4: Trend in key BU control indicators from 2006 to 2014. During this period, the ulcerative form of BU constituted about 83% of lesions and 90% of all lesions were located on the limbs. On average, children below 15 years of age constituted 45% of cases. The proportion of category 3 lesions rose from 9% in 2008 to 52% in 2014 and the proportion of BU cases confirmed by PCR dropped from 57% in 2010 to 20% in 2014.
(XLSX)

S3 Dataset For Fig 5: Evolution of confirmed BU endemic health districts between 2001 and 2014. During the period 2001–2002 there were only two known BU endemic health districts in the Nyong basin of central Cameroon [19]. The national BU survey in 2004 revealed the presence of BU in 19 other health districts [20]. From 2006–2014, treated BU cases originated from sixty-four health districts.
(XLSX)

S1 File Compressed shape files _Cameroon health district: The Cameroon health district shape files used for drawing up of the maps in this article were obtained from the Sub Department for epidemiological surveillance in the Ministry of Public Health, Cameroon. (RAR)

S1 Survey Report of the preliminary survey on the situation of Buruli ulcer in Cameroon carried out by Um Boock and collaborators in 2004. (PDF)
