# Correction: Buruli Ulcer in Cameroon: The Development and Impact of the National Control Programme

**DOI:** 10.1371/journal.pntd.0004438

**Published:** 2016-02-01

**Authors:** The PLOS Neglected Tropical Diseases Staff

There are errors in the author affiliations. The affiliations should appear as shown here: Earnest Njih Tabah^1,2,3,4^, Dickson Shey Nsagha^5^, Anne-Cécile Zoung-Kanyi Bissek^4,6^, Alfred Kongnyu Njamnshi^4,7^, Martin W. Bratschi^2,3^, Gerd Pluschke^2,3^, Alphonse Um Boock^8^

**1** National Yaws, Leishmaniasis, Leprosy and Buruli ulcer Control Programme, Ministry of Public Health, Yaounde, Cameroon, **2** Swiss Tropical and Public Health Institute, Basel; University of Basel, Basel, Switzerland, **3** The University of Basel, Basel, Switzerland, **4** Faculty of Medicine and Biomedical Sciences, The University of Yaounde 1, Yaounde, Cameroon, **5** Department of Public Health and Hygiene, Faculty of Health Sciences, University of Buea, Cameroon, **6** Department of Operational Research in Health, Ministry of Public Health, Yaounde, Cameroon, **7** Department of Neurology, Central Hospital, Yaounde, Cameroon, **8** Regional Bureau for Africa of the FAIRMED Foundation, Yaounde, Cameroon.
