# Supplementary material for: Buruli Ulcer in Cameroon: The Development and Impact of the National Control Programme
Source: PLoS Negl Trop Dis. 2016 Jan 13;10(1):e0004224. doi: 10.1371/journal.pntd.0004224 (PMC4711896; doi:10.1371/journal.pntd.0004224)
Supplement: S1 Survey — (PDF) [file pntd.0004224.s005.pdf]

Lepra.ch
Aide aux Lépreux Emmaüs-Suisse

Swiss Tropical Institute
Institut Tropical Suisse
Schweizerisches Tropeninstitut

Swiss Centre for
International Health

ENQUETE PRELIMINAIRE NATIONALE SUR LA SITUATION DE

L'ULCERE DE BURULI AU CAMEROUN

Aide aux Lépreux Emmaüs Suisse

Institut Tropical Suisse

&
Ministère de la Santé Publique

Dr Um Boock Alphonse :
Représentant régional pour l'Afrique
Aide aux Lèpreux Emmaüs Suisse

Dr Sarah Eyango : Centre Pasteur du Cameroun

Dr Kaspar Wyss : Institut Tropical Suisse de Bâle

Dr Nsom Mba Charles

Mars –novembre 2004

Financement : ALES

Table des matières

1. REMERCIEMENTS 3

2. LISTE DES ABREVIATIONS 4

3. RESUME 5

4. SITUATION GEOGRAPHIQUE DU CAMEROUN 9

5. CONTEXTE 10

6. METHODOLOGIE 11

6.1 ZONE D'ETUDE 11
6.2 DEFINITION DES CAS 15
6.3 DEROULEMENT DE L'ENQUETE ET COLLECTE DES DONNEES 17
6.4 ANALYSES DE LABORATOIRE 17
6.5 ANALYSE DES DONNEES STATISTIQUES 18
7. RESULTATS

8. COMMENTAIRES 22

9. CONCLUSION 25

10. ANNEXES 26

10.1 PROVINCE DE L'ADAMAOUA : 26
10.2. PROVINCE DU CENTRE 27
10.3. PROVINCE DE L'EST : 28
10.4. PROVINCE DE L'EXTREME-NORD 28
10.5. PROVINCE DU SUD 28
10.6. PROVINCE DU SUD-OUEST : 29

1. REMERCIEMENTS

Nous adressons nos vifs remerciements à toute l'équipe de santé d'Ayos dont l'apport a été très déterminant notamment dans l'examen des patients . Il s'agit tout particulièrement de : Dr Binzouli , Chef de Service de santé de district d'Ayos , Mr Kombang Thomas , Mr Ntoungou Serge , Mr Samentanga Menta et Mr Ze Bekolo , tous en service à l'hôpital de district d'Ayos.

Nos remerciement vont aussi à Dr Ngos François, Chef de service de Santé de District d'Akonolinga, pour sa participation à cette enquête.

Nous remercions tout particulièrement tous les Délégués provinciaux de la santé et les Superviseurs lèpre provinciaux pour leur contribution à la réalisation de cette étude .

Nous remercions le Centre Pasteur de Yaoundé sans qui la confirmation biologique des cas n'aurait pas été possible .

Nous remercions également les Dr Nicolaus Lorenz et Kaspar Wyss de l'Institut Tropical Suisse de Bâle .

Nous voulons enfin remercier toutes les personnes des différents villages qui ont volontairement et spontanément acceptées de sacrifier un peu de leur temps afin de contribuer , chacun à sa manière à la production de toutes les données qui constituent le socle du présent travail.

Nous exprimons enfin notre gratitude à toutes les personnes qui de près ou de loin ont contribué à la réalisation de cette étude .

2. LISTE DES ABREVIATIONS

ADN : Acide désoxyribonucléique
ALES : Aide aux Lèpreux Emmaüs Suisse
BAAR : Bacille Acido Alcoolo Résistant
BCG : Bacille Calmette et Guérin
CSI : Centre de Santé Intégré
CPC : Centre Pasteur du Cameroun
ITS : Institut Tropical Suisse
MSP : Ministère de la Santé
NDVI : Normalized Difference Vegetation Index
PCR : Polymerase chain reaction
OMS : Organisation Mondiale de la santé
UB : Ulcère de Buruli

3. RESUME

L'Ulcère de Buruli (UB) est une maladie infectieuse provoquée par le *Mycobacterium ulcerans*. Après la tuberculose et la lèpre, elle représente l'affection humaine à mycobactérie la plus fréquemment rencontrée. [i,ii] L'agent pathogène a été décrit pour la première fois par MacCallum et coll. en Australie en 1948 [iii], bien que probablement déjà décrit par Sir Robert Cook en Uganda en 1887.

Des foyers endémiques ont été rapportés dans des zones rurales intertropicales dans plus de 30 pays en Afrique, en Asie, en Océanie, en Amérique du Sud et aussi au Mexique et en Australie.

Au Cameroun, la maladie a été décrite pour la première fois par Ravisse et coll. en 1975 et Boisvert en 1977 dans un foyer très localisé dans la vallée du Nyong entre les villes d'Ayos et d'Akonolinga (Province de Centre). [iv, v, vi]

Une enquête épidémiologique menée en 2001 dans le bassin du Nyong sous la direction de l'Aide aux Lépreux Emmaüs – Suisse (ALES), a permis de classer le Cameroun parmi les pays endémiques d'Afrique et l'ampleur de la maladie comme un réel problème de santé publique.

Depuis lors, plusieurs partenaires dont l'ALES, les Médecins Sans Frontière Suisse et le Centre Pasteur du Cameroun, se sont engagés à appuyer techniquement et financièrement le pays pour la prise en charge qui reste limitée dans la province du Centre dans les districts d'Ayos et Akonolinga.

Cependant, le personnel de santé continuait à rapporter sporadiquement des cas suspects de l'Ulcère de Buruli dans différentes régions du pays telles que les Provinces de l'Extrême-Nord, du Sud-Ouest et du Centre ; ces données sont restées sans confirmation diagnostique. [vii]

Ces observations on conduit l'ALES et le Ministère de la Santé du Cameroun avec l'appui technique de l'Institut Tropical Suisse à lancer une enquête nationale qui s'est déroulée, entre le mois de mars et novembre 2004. La première phase de ce travail a consisté à identifier des zones suspectes de la maladie. L'investigation de la suspicion des cas s'est faite sur base d'un questionnaire qui a été administré dans 64% (n=98) des districts de santé, à travers le réseau lèpre qui reste le plus étendu et le plus structuré du système de santé. Les résultats de cette première phase ont montré que les 10 provinces que compte le pays sont suspectes de la maladie.

La deuxième phase de l'enquête dont le but était de rechercher d'autres foyers en dehors de celui d'Ayos et Akonolinga , a permis de connaître la prévalence de la maladie dans certaines zones suspectes à travers la confirmation bactériologique et clinique des cas d'ulcère de Buruli.

La présente enquête prospective a couvert toutes les zones suspectées lors de la première phase ; d'autres villages ont été identifiés lors de l'examen des malades. Un plan de travail préétabli en accord avec les Contrôleurs lèpre de District, les chefs de village et de Centres de santé intégrés a servi de base pour l'organisation du travail sur le terrain. Les populations étaient sensibilisées sur l'arrivée des enquêteurs au moins 24 heures avant. Une session d'éducation sanitaire portant sur l'ulcère de

Buruli commençait toujours les travaux, après avoir bien expliqué les buts de notre présence dans le village.

Tous les patients qui répondaient à notre définition de cas étaient inclus dans l'enquête comme cas d'ulcère de Buruli et leurs prélèvements soumis à la bactériologie pour la recherche de Mycobactérium Ulcerans. Ils ont été interrogés et examinés cliniquement. Une fiche préalablement apprêtée pour l'enquête a été remplie pour chaque cas ; cette fiche portait les informations suivantes : identité du malade, présence ou non des autres cas d'ulcère de Buruli dans la famille, notion d'exposition aux facteurs de risque et antécédents de vaccination par le BCG sur la base de la présence de la cicatrice vaccinale sur la face intérieure de l'avant-bras gauche ou une preuve de vaccination (carte vaccinale) . Après avoir pris la taille et le poids, chaque patient a eu un examen dermatologique détaillé destiné à répertorier et à décrire les caractéristiques des lésions dues à l'ulcère de Buruli en particulier.

La description et la localisation des lésions ainsi que les séquelles éventuelles ont été notées sur la fiche technique. La localisation des lésions a également été reportée sur un schéma du corps. La forme clinique de l'ulcère de Buruli définie en forme active ou inactive et leur différents types selon les recommandations de l'OMS ont été également enregistrés.

Des prélèvements réalisés par l'écouvillonnage ont été faits pour tous les malades présentant un ulcère actif. Ces prélèvements ont été effectués sous les bords décollés de l'ulcère. Un frottis a été systématiquement confectionné après chaque prélèvement pour recherche de BAAR après coloration par la technique de coloration de Ziehl-Neelsen au Centre Pasteur de Yaoundé. Deux écouvillons par malade ont été systématiquement conservés à + 4° et envoyé au Centre Pasteur de Yaoundé , laboratoire de référence pour analyse de confirmation bactériologique par la technique PCR (polymerase chaine reaction) qui utilise des amorces publiées (MU5 et MU6) de l'IS2404 pour détecter l'ADN de M. Ulcerans et par culture après une extraction d'ADN par le kit d'extraction otagene.

Les nodules suspectés ont été systématiquement excisés et un fragment retenu pour l'anatomie pathologie et un autre à la bactériologie pour confirmation .

Tous les patients ayant un ulcère ont eu un pansement de celui-ci et du petit matériel de pansement.

La saisie et l'analyse des données s'est faite sur Epi Info 6.0 . Le test de chi carré a été utilisé pour préciser les associations entre des variables dichotomiques. Les variables continues ont été analysées à l'aide du test de T student .

1525 patients répartis dans 167 villages ont été examiné . 705 cas d'ulcère de Buruli (formes évolutives et formes inactives confondues) ont été enregistrés donc 84% (n=590) cas évolutifs confirmés contre 16 % (n= 115) cas non évolutifs.

A l'exception des provinces du Nord, du Littoral , de l'Ouest et du Nord-Ouest, la présence de la maladie a été confirmée dans les six autres provinces : Adamaoua , Centre , Sud , Sud-Ouest , Est et Extrême-nord . Le district de santé de Mbalmayo dans la province du Centre s'avère être aussi un grand foyer de la maladie .

La province du Centre hors mis les districts de santé d'Ayos et Akonolinga reste la plus endémique avec 49% (n= 342) de tous les cas enregistrés suivi de la province de l'Est 32% (n= 229) et celle du Sud-Ouest 11% (n= 76).

La différence entre la tranche d'âge comprises entre 0 à 15 ans (n=346) et celles supérieures à 15 ans (n= 359) n'est pas statistiquement significative (P>0.05) Les hommes (52%) sont sensiblement plus atteints que les femmes (48 %).

Plus de 75% (n=443) des lésions évolutives rencontrées sont au stade d'ulcère et jusqu'à 5% (n= 30) parmi elles sont au stade d'ostéomyélite. Les formes précoces (nodules) ne représentent qu'environ 2% (n= 12).

Notre étude montre que 52 % (n= 369) des lésions se trouvent au membre inférieur . Les répartitions des localisations hommes - femmes ne sont pas statistiquement significatives (p > 0.05)

Le délai moyen d'évolution de lésions depuis l'apparition était de 10,3 mois (intervalle d'un mois à 5 ans) avec une médiane de 5 mois, ce qui témoigne de la chronicité des lésions.

Environ 93 % (n=549) des patients avec des lésions actives ont eu au moins une fois recours à la médecine traditionnelle contre 21 % (n=124) pour la médecine moderne.

La cicatrice vaccinale BCG n'a été observée que chez environ 50 % (n= 295) des malades avec des lésions évolutives.

Nous n'avons pas jugé utile de mener l'enquête dans les districts de santé d'Akonolinga et Ayos par ce que les populations étaient assez bien sensibilisées et la prise en charge biomédicale aussi bien organisée et surtout ce sont des anciens foyers. Toutefois, nous avons considéré les cas confirmés (n= 225) et enregistrés dans ces districts au cours de l'année 2004 dans les données de la prévalence nationale.

La prévalence de la maladie en tenant compte des districts d'Ayos et Akonolinga est de 930 malades dont environ 73% (n= 676) des cas sont des formes évolutives .

Il est fort probable que cette prévalence soit sous estimée à cause des moyens limités et l'inaccessibilité dans certaines zones potentiellement endémiques à cause de l'état des routes en saison pluvieuse. A ces raisons s'ajoutent la mauvaise collaboration avec les tradi-praticiens, les interdictions imposées à certains malades par les guérisseurs et le scepticisme de la population envers la médecine moderne jugée d'inefficace.

Si les caractéristiques des foyers trouvés dans la province du centre sont similaires à ceux décrit dans la littérature , c'est à dire dans des zones marécageuses inondables il n'en va pas de même de ceux de la province de l'Adamaoua qui mérite une clarification.

Pour ce qui est des cas non évolutifs , on constate que l'ulcère de Buruli est une importante source de déficits de mobilité entraînant très souvent des handicaps plus ou moins importants. Les causes des déficits de mobilité sont pour la plupart directement liées à une absence de rééducation appropriée. Elles sont le plus souvent

le résultat d’un manque de mobilisation et/ou d’un positionnement incorrect tout au long du processus de guérison de la plaie et de sa cicatrisation.

Les rétractions et adhérences cutanées représentent la première cause des déficits de mobilité.

Près de 80% des troubles fonctionnels constatés étaient secondaires aux problèmes cutanés. Sans rééducation, les résultats fonctionnels concernant les membres supérieurs semblent aussi médiocres après chirurgie qu’après une guérison spontanée ou un traitement par la médecine traditionnelle.

La lutte contre les rétractions cutanées demande une rééducation spécifique similaire à celle des grands brûlés.

Du fait des limites de cette étude préliminaire, des études régionales plus ciblées méritent encore d’être menées afin de connaître la prévalence réelle de la maladie au Cameroun. Toutefois les données actuelles constituent une bonne base pour le développement du programme national de lutte contre cette maladie.

4. SITUATION GEOGRAPHIQUE DU CAMEROUN

Pays d'Afrique Centrale, le Cameroun est situé au fond du Golf de Guinée, entre les 2ème et 3ème de latitude Nord et les 9ème et 16ème degrés de longitude Est. De forme triangulaire, le pays s'étire en hauteur, du sud au Lac Tchad, sur près de 1 200 km ; la base s'étale d'Ouest et Est sur 800 km, présentant ainsi une superficie d'environ 472 000 km². Le Cameroun est limité à l'Est par le Tchad et la République Centrafricaine, au Sud par le Congo, le Gabon et la Guinée Equatoriale, au Sud-ouest par l'Océan Atlantique, et à l'Ouest par le Nigeria. Enfin, au sommet supérieur du triangle, au Nord, il est coiffé par le Lac Tchad.

Le milieu naturel du Cameroun est diversifié. On dit de ce pays qu'il est « l'Afrique en miniature ». En effet, plusieurs types de régions naturelles contribuent à la diversité géographique du pays :

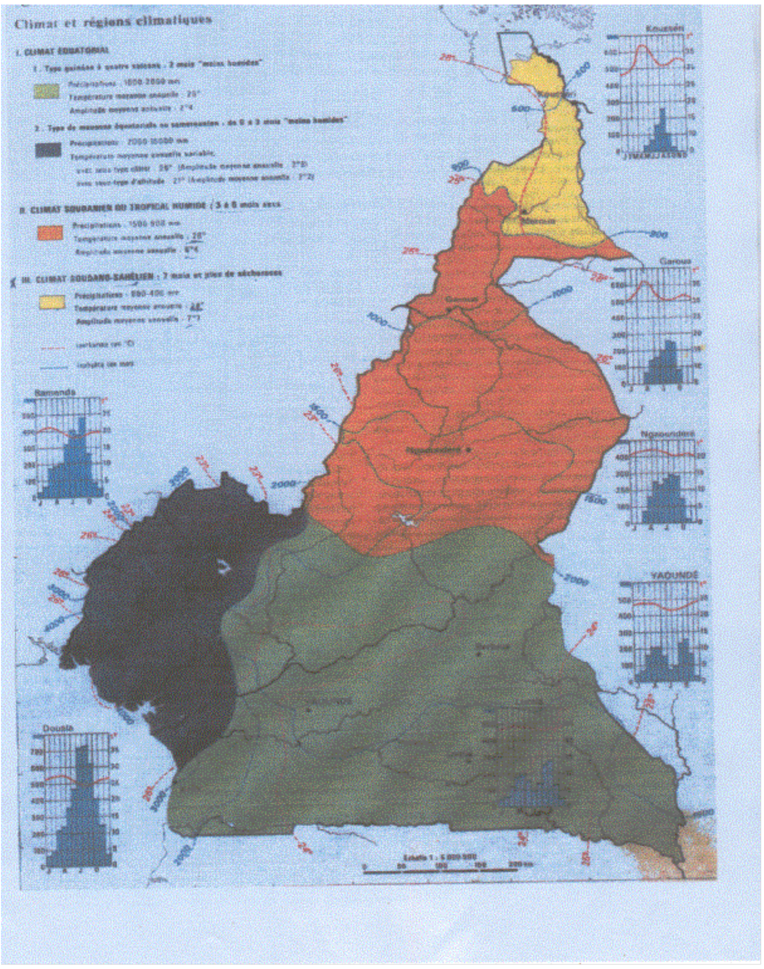

- Le sud forestier (provinces du Centre, de l'Est, du Littoral, du Sud et du Sud-ouest) se situe dans des zones maritimes et équatoriales et se caractérise par une végétation dense, un vaste réseau hydrographique et un climat chaud et humide aux précipitations abondantes. Cette région est propice à la culture du cacao, du palmier à huile , de la banne, de l'hévéa, du tabac... Elle abrite les deux plus grandes villes du pays : Douala (principal port maritime et capitale économique avec ses activités commerciales et industrielles) et Yaoundé (deuxième ville et capitale politique et administrative). D'importants centres urbains comme Edéa, caractérisé par son industrie lourde et sa centrale

hydroélectrique, et Limbe, siège de l'industrie pétrolière, font également partie de cette région.

- Les hauts plateaux de l'Ouest (provinces de l'Ouest et du Nord-ouest), dont l'altitude moyenne est supérieure à 1 100 m, forment une région riche en terres volcaniques favorables à l'agriculture (café, maraîchers…). La végétation y est moins dense que dans le sud forestier et le climat frais qui y règne est favorable à l'éclosion de toutes sortes d'activités. la forte densité de peuplement par rapport à la moyenne nationale en fait une des premières zones d'émigration. Les principales villes sont Bafoussam et Bamenda.

- Le nord soudano sahélien (provinces de l'Adamaoua, du Nord et de l'Extrême nord) est une région de savanes et de steppes. En dehors du plateau de l'Adamaoua où le climat est plus tempéré, le reste de cette région est caractérisé par un climat tropical chaud et sec aux précipitations de plus en plus limitées au fur et à mesure que l'on se rapproche du Lac Tchad. La région est propice à l'élevage du bovin et à la culture du coton et du mil.

5. CONTEXTE

L'Ulcère de Buruli (UB) est une maladie infectieuse causée par le *Mycobacterium ulcerans*. Après la tuberculose et la lèpre, elle représente l'affection humaine à mycobactérie la plus fréquemment rencontrée. [viii,ix] L'agent pathogène a été décrit pour la première fois par MacCallum et coll. en Australie en 1948 [x], bien que probablement déjà décrit par Sir Robert Cook en Uganda en 1887.

Depuis lors, des foyers endémiques ont été rapportés dans des zones rurales intertropicales dans plus de 30 pays en Afrique, en Asie, en Océanie, en Amérique du Sud et aussi au Mexique et en Australie. A ce jour l'Afrique de l'ouest est la région la plus affectée avec des taux de prévalence de 16% [xi] dans quelques communautés au Côte d'Ivoire. Au Ghana où la maladie est endémique dans tout le pays, une prévalence même plus de 20% a été estimée dans une communauté.[xii]

La forte recrudescence observée depuis 1980 notamment en Afrique de l'Ouest a emmené l'OMS a reconnaître l'Ulcère de Buruli en 1998 comme une importante maladie émergente et a lancé une « Initiative mondiale contre l'Ulcère de Buruli ».

Au Cameroun, la maladie a été décrite pour la première fois par Ravisse et coll. en 1975 et Boisvert en 1977 dans un foyer très localisé dans la vallée du Nyong entre les villes d'Ayos et d'Akonolinga (Province de Centre). [xiii, xiv, xv]

Une enquête épidémiologique réalisée en 2001 dans le bassin du Nyong et dirigée par l'Aide aux Lépreux Emmaüs – Suisse (ALES), a permis de classer le Cameroun parmi les pays endémiques d'Afrique et l'ampleur de la maladie comme un réel problème de santé publique.

Depuis 2001, plusieurs partenaires dont l'ALES, les Médecins Sans Frontière Suisse et le Centre Pasteur de Cameroun, se sont engagés à appuyer techniquement et financièrement le pays pour la prise en charge des cas de cette maladie.

Au cours de l'année 2003 des efforts importants de structuration ont été réalisés dans le programme national y compris une enquête sur la connaissance, les attitudes et les pratiques des populations dans la vallée de Nyong afin de définir des stratégies d'interventions appropriées et adaptées. [35,36] Les résultats de ces travaux confirment, entre autre que la maladie est associée à des sanctions , à une violation des normes sociales , que le rôle des tradi-praticiens est crucial et doit avoir des implications dans l'organisation de la prise en charge.

Par ailleurs, le personnel de santé rapporte sporadiquement des cas suspects de l'Ulcère de Buruli dans différentes régions du pays telles que les Provinces de l'Extrême-Nord, du Sud-Ouest et du Centre ; ces données n'ont jamais fait partie des statistiques sanitaires de routine et sont restées sans confirmation diagnostique. [xvi]

Ces observations on conduit l'ALES et le Ministère de la Santé du Cameroun avec l'appui technique de l'Institut Tropical Suisse à lancer une enquête nationale qui s'est déroulée, entre le mois de mars et novembre 2004. La première phase de ce travail a consisté à identifier des zones suspectes de la maladie. L'investigation de la suspicion des cas s'est faite sur base d'un questionnaire qui a été administré sur le terrain dans 98 (64%) districts de santé, à travers le réseau lèpre qui reste le plus étendu et le plus structuré du système de santé. Les résultats de cette première phase ont montré que les 10 provinces que compte le pays sont suspectes de la maladie.

La deuxième phase de l'enquête a consisté à la description clinique selon la définition de l'OMS et à la confirmation bactériologique des prélèvements sur des cas suspects.

6. METHODOLOGIE

6.1 Zone d'étude

L'enquête a couvert toutes les 10 provinces du pays. Les districts, les aires de santé et villages ont été identifiés lors de la première phase de l'enquête (Anja S. , Kaspar W., Alphonse U.B.) ; d'autres villages ont été identifiés lors de l'examen des malades.

De manière opérationnelle , nous entendons par zone suspecte , un espace géographique bien délimité dans lequel il y'a des soupçons de l'existence des cas d'ulcère de Buruli à partir des informations recueillies dans les fiches d'enquêtes remplies dans les formations sanitaires. Cette définition ne tient pas compte de l'écosystème du lieu concerné.

On parle de présence de la maladie dans une zone s'il y 'a au moins un cas cliniquement et bactériologique ment confirmés dans cet espace.

Figure 7. Distribution des régions suspectes et la température annuelle moyenne du terrain

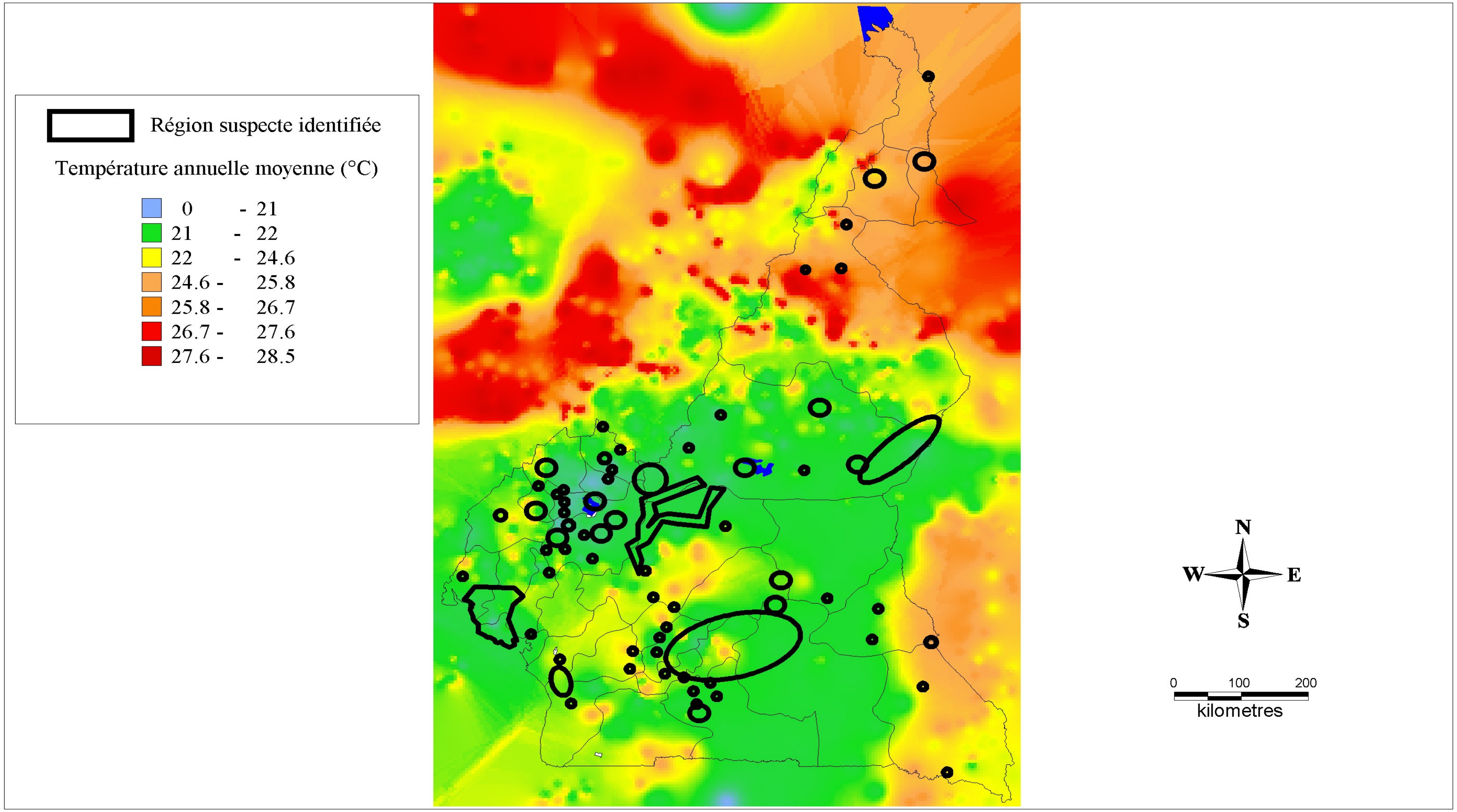

Figure 2. Distribution des régions suspectes et de la végétation (NDVI)

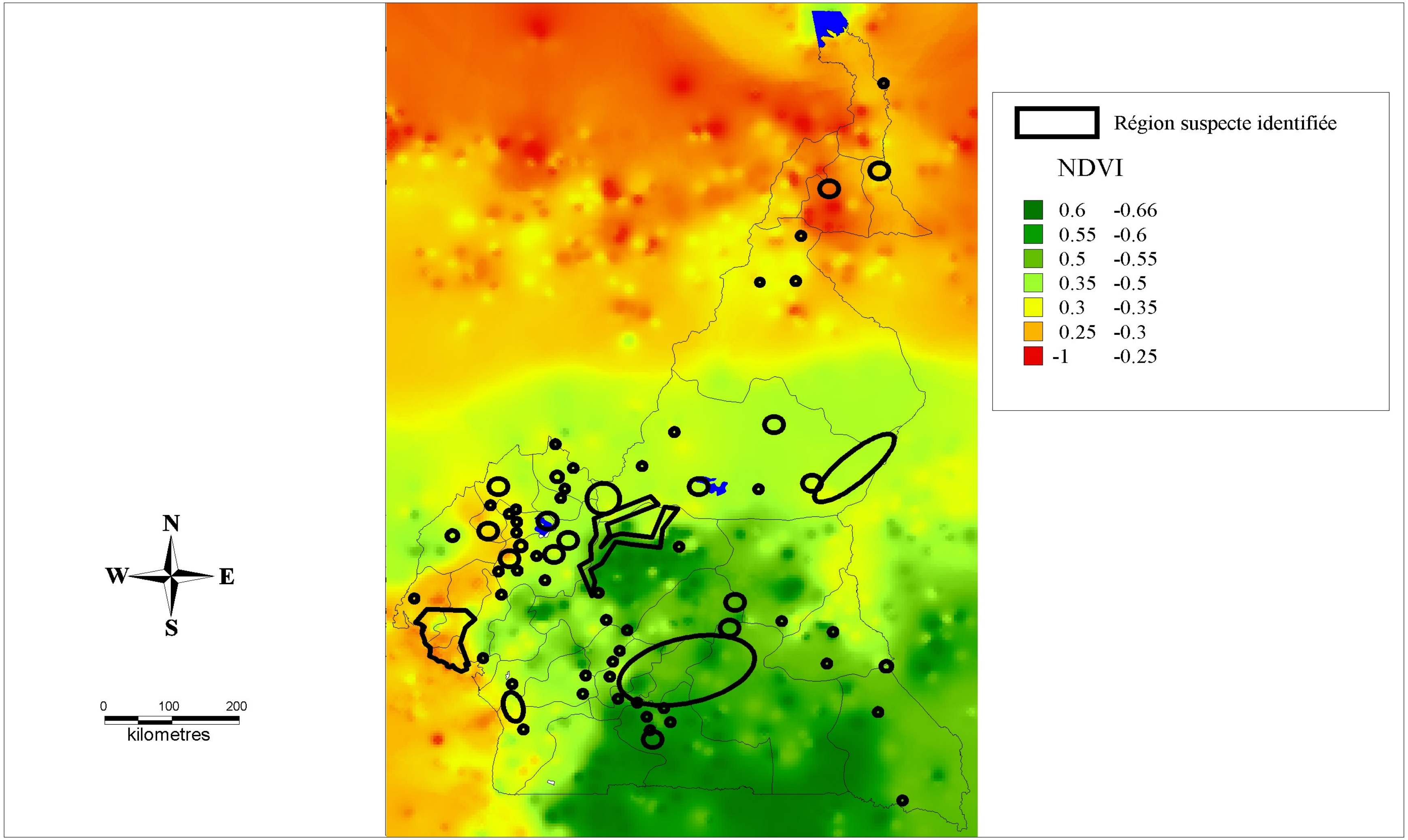

Figure 3. Régions suspectes par rapport à l'hydrographie et régions suspectes identifiées

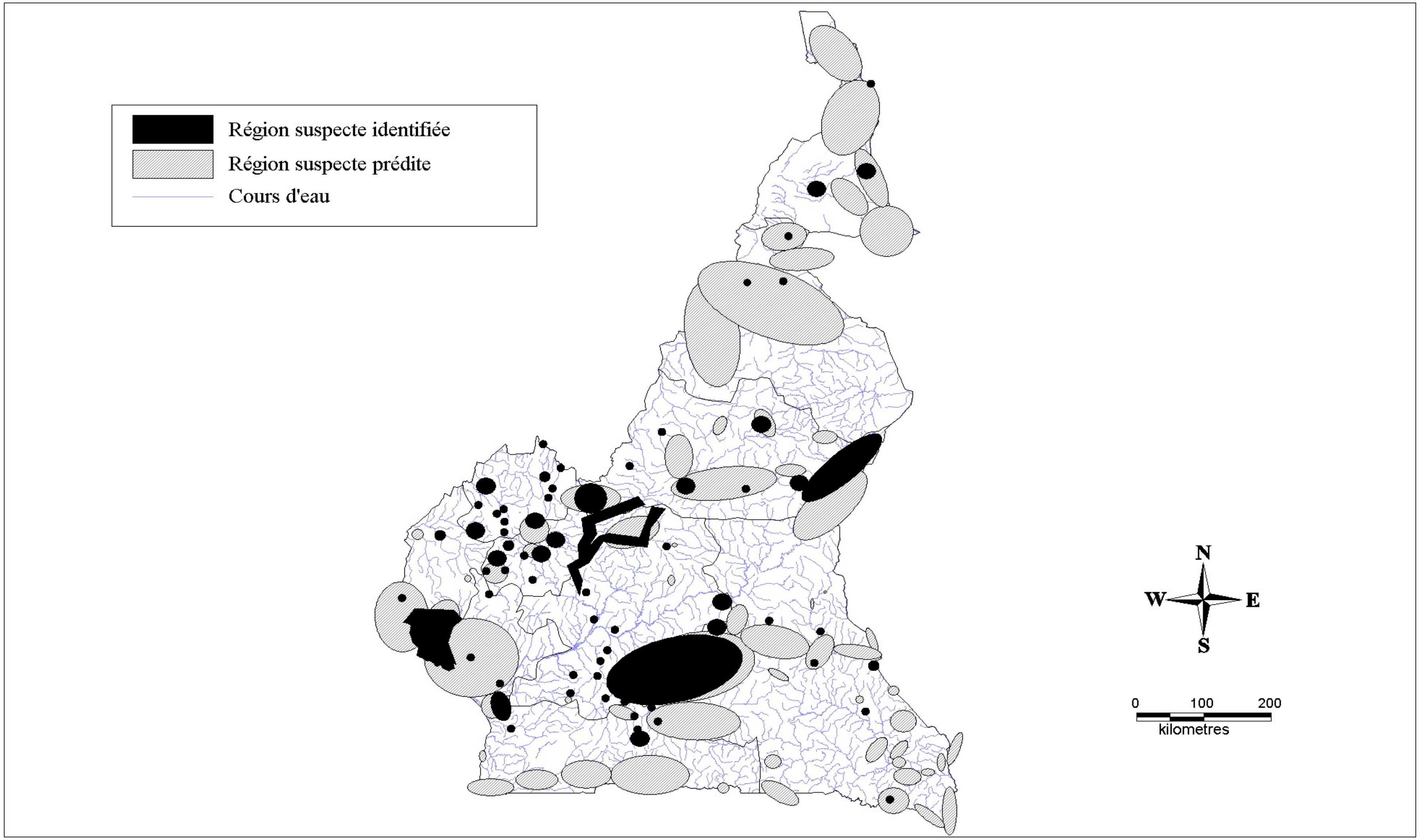

6.2 Définition des cas

Tous les cas qui répondaient aux critères suivants étaient inclus dans l'enquête comme cas d'ulcère de Buruli ; leurs prélèvements étaient systématiquement soumis à la bactériologie pour recherche de Mycobatérium Ulcerans :

Ulcère de Buruli, forme évolutive :

1. Nodule : c'est un lésion s'étendant de la peau au tissu cutané ; elle a un diamètre souvent compris entre 1 à 2 cm . Elle est en général indolore .

Photo n1 : oedème

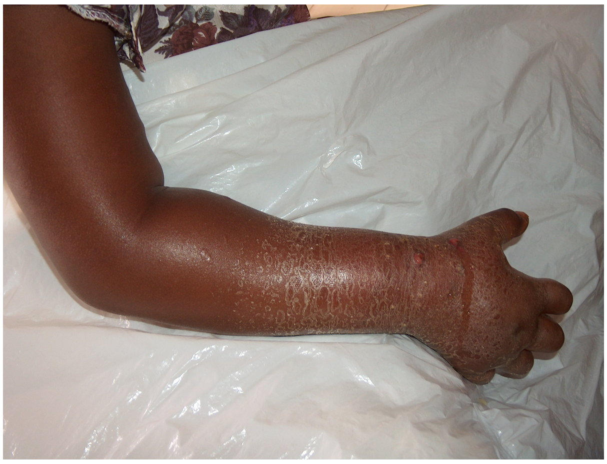

2. Œdème : tuméfaction ferme , indolore, diffuse , étendue ne prenant pas le godet . La zone touchée a des bords mal définis.

3. Plaque : Lésion ferme, indolore bien démarquée , surélevée , de plus de 2 cm de diamètre et à bord irréguliers. Au dessus de la lésion , la peau est souvent rouge ou dépigmentée.

Photo n°2 : lésion ulcéreuse

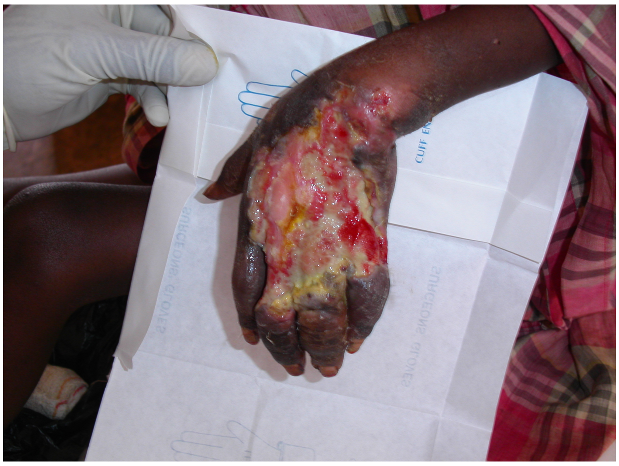

4. Ulcère : se caractérise par un centre nécrosé, des bords creusés et une peau en périphérie de l'ulcère infiltrée et hyper pigmentée .

Ou Ulcère en voie de cicatrisation consistent avec l'UB, i.e. épidermisation et cicatrisation sur un fond affaissé, débutant à partir des bords apparemment dévitalisés

Photo n° 3 : ulcère en voie de cicatrisation

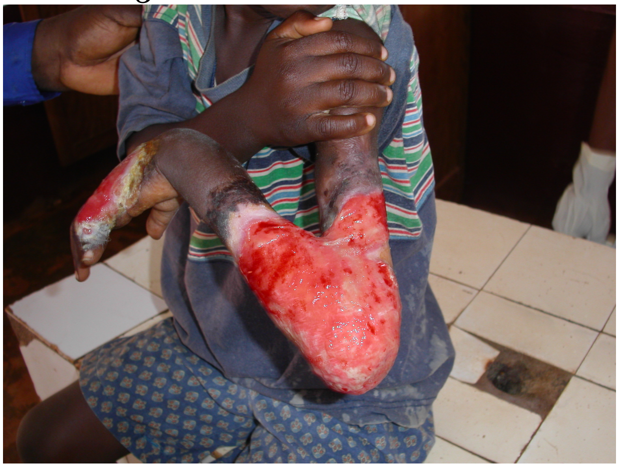

Ulcère de Buruli, forme inactive :

Photo n°4 : Séquelle due à l'ulcère de Buruli

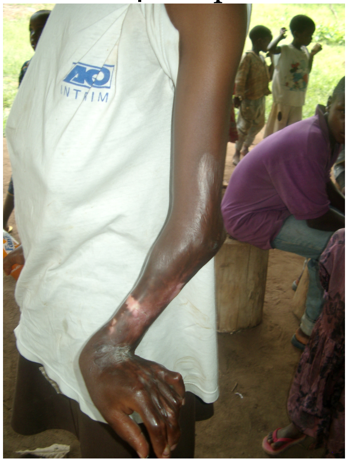

Cicatrice affaissée en étoile, avec ou sans séquelles

6.3 Déroulement de l'enquête et collecte des données

Les villages dans lesquels des cas avaient été suspectés lors de la première phase de l'enquête ont été visités selon un plan préétabli en accord avec les Contrôleurs lèpre de District, les chefs de village et de Centres de santé intégrés. Les populations étaient chaque fois largement sensibilisées sur l'arrivée des enquêteurs au moins 24 heures avant.

Les enquêteurs s'installaient chaque fois soit dans le centre/case de santé soit dans la maison du chef du village ou d'une élite déjà préparée pour les besoins de l'enquête. Une session d'éducation sanitaire portant sur l'ulcère de Buruli commençait toujours les travaux, après avoir bien expliqué les buts de notre présence dans le village.

Tous les patients inclus ont été interrogés et examinés cliniquement. Une fiche préalablement apprêtée pour l'enquête a été remplie pour chaque cas ; cette fiche portait les informations suivantes : identité du malade, présence ou non des autres cas d'ulcère de Buruli dans la famille, notion d'exposition aux facteurs de risque et antécédents de vaccination par le BCG sur la base de la présence de la cicatrice vaccinale sur la face intérieure de l'avant-bras gauche ou une preuve de vaccination (carte vaccinale) . Après avoir pris la taille et le poids, chaque patient a eu un examen dermatologique détaillé destiné à répertorier et à décrire les caractéristiques des lésions dues à l'ulcère de Buruli en particulier.

La description et la localisation des lésions ainsi que les séquelles éventuelles ont été notées sur la fiche technique. La localisation des lésions a également été reportée sur un schéma du corps. La forme clinique de l'ulcère de Buruli définie en forme active ou inactive et leur différents types selon les recommandations de l'OMS ont été également enregistrés.

Tous les patients ayant un ulcère ont eu un pansement de celui-ci et du petit matériel de pansement.

6.4 Analyses de laboratoire

Des prélèvements réalisés par l'écouvillonnage ont été faits sur un malade sur quatre choisi au hasard et présentant un ulcère actif. Ces prélèvements ont été effectués sous les bords décollés de l'ulcère. Un frottis a été systématiquement confectionné après chaque prélèvement pour recherche de BAAR après coloration par la technique de coloration de Ziehl-Neelsen au Centre Pasteur de Yaoundé. Si possible deux écouvillons par malade ont été systématiquement conservés à + 4° et envoyé au Centre Pasteur de Yaoundé , laboratoire de référence pour analyse de confirmation bactériologique par la technique PCR (polymerase chaine reaction) qui utilise des amorces publiées (MU5 et MU6) de l'IS2404 pour détecter l'ADN de M. Ulcerans et par culture après une extraction d'ADN par le kit d'extraction otagene.

Les nodules suspectés ont été systématiquement excisés et un fragment retenu pour l'anatomie pathologie et un autre à la bactériologie pour confirmation .

6.5 Analyse des données statistiques

La saisie et l'analyse des données s'est faite sur Epi Info 6.0 . Le test de chi carré a été utilisé pour préciser les associations entre des variables dichotomiques. Les variables continues ont été analysées à l'aide du test de T student .

7. RESULTATS

A l'exception des provinces du Nord, du Littoral , de l'Ouest et du Nord-Ouest, la présence de la maladie a été confirmée dans les six autres provinces : Adamaoua , Centre , Sud , Sud-Ouest , Est et Extrême-nord .

1525 patients répartis dans 167 villages ont été examiné . Environ 42 % (n=638) des patients examinés ne répondaient pas cliniquement à la définition des cas selon l'OMS et n'ont pas été retenus pour la suite de l'enquête.

13% (n=167) des prélèvements sur des lésions actives se sont avérés négatifs à l'examen bactériologique.

6% (n= 15) des cas cicatrisés examinés ne correspondaient pas aux cicatrices post ulcère de Buruli sur la base de notre expérience sur la maladie.

Au total, 705 cas d'ulcère de Buruli (formes évolutives et formes inactives confondues) ont été enregistrés donc 84% (n=590) cas évolutifs confirmés contre 16 % (n= 115) cas non évolutifs.

Fig. 1 : distribution par sexe et par âge

GROUPE D'AGE	SEXE MASCULIN	SEXE FEMININ	TOTAL	%*
0 à 5 ans	41	43	84	12%
6 à 10 ans	68	53	121	17%
11 à 15 ans	78	63	141	20%
16 à 20 ans	58	53	111	16%
Plus de 20 ans	119	129	248	35%
**TOTAL**	**364**	**341**	**705**	**100%**

L'enquête révèle que la différence entre la tranche d'âge entre 0 à 15 ans (n=346) et celle supérieure à 15 ans (n= 359) n'est pas statistiquement significative (P>0.05) Les hommes (52%) sont sensiblement plus atteints que les femmes (48 %).

La province du Centre hors mis les foyers d'Ayos et Akonolinga reste la plus endémique avec 49% (n= 342) de tous les cas enregistrés suivi de la province de l'Est 33% (n=229) et la province du Sud-Ouest vient en troisième position 11% (n= 76).

Fig n°2 : distribution des cas identifiés selon les provinces

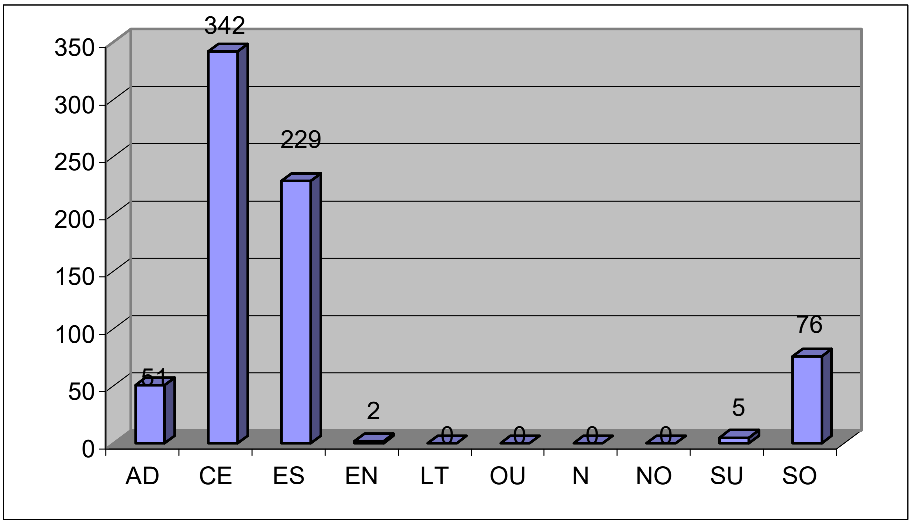

Plus de 75% (n=443) des lésions évolutives sont au stade d'ulcère et jusqu'à 5% (n= 30) parmi elles sont au stade d'ostéomyélite. Les formes précoces (nodules) ne représentent qu'environ 2% (n= 12).

Fig.3: Formes cliniques

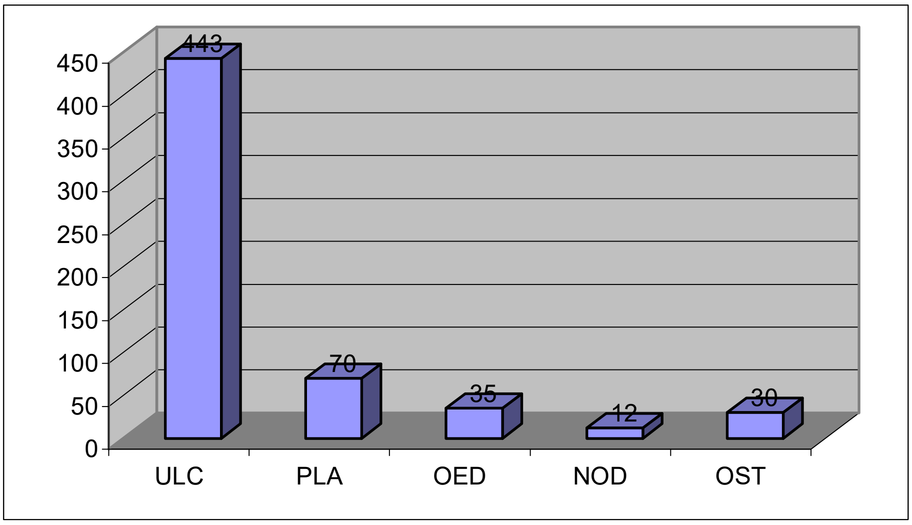

Notre étude montre que 52 % (n= 369) des lésions se trouvent au membre inférieur . Les répartitions des localisations hommes - femmes ne sont pas statistiquement significatives ($p > 0.05$)

Tableau 4. Localisation anatomique des lésions

Localisation	0 – 15 ans		15 ans et plus		Total
	Homme	Femme	Homme	Femme	
Tête, cou	9	5	2	1	17
Tronc	5	7	1	3	16
Membre supérieur	80	68	79	78	305
Membre inférieur	93	79	95	102	369

Le délai moyen d'évolution de lésions depuis l'apparition était de 10,3 mois (intervalle d'un mois à 5 ans) avec une médiane de 5 mois, ce qui témoigne de la chronicité des lésions.

Environ 93 % (n=549) des patients avec des lésions actives ont eu au moins une fois recours à la médecine traditionnelle contre 21 % (n=124) pour la médecine moderne.

La cicatrice vaccinale BCG n'a été observée que chez environ 50 % (n= 295) des malades avec des lésions évolutives.

Nous n'avons pas jugé utile de mener l'enquête dans les districts de santé d'Akonolinga et Ayos par ce que les populations étaient assez bien sensibilisées et la prise en charge biomédicale aussi bien organisée et surtout ce sont des anciens foyers. Toutefois, nous avons considéré les cas confirmés (n= 225) et enregistrés dans ces districts au cours de l'année 2004 dans les données de la prévalence nationale.

La prévalence de la maladie en tenant compte des districts d'Ayos et Akonolinga est de 930 malades dont environ 73% (n= 676) des cas sont des formes évolutives .

8. COMMENTAIRES

L'enclavement , aggravé par le mauvais état des routes en saison pluvieuse et le manque de chaîne de froids dans la plupart des villages , ne nous ont pas toujours permis de transférer les prélèvements à temps au Centre Pasteur de Yaoundé pour examen bactériologique ; beaucoup de prélèvements se sont donc détériorés ou simplement se sont négativés. Quelques cas douteux sur le plan clinique ont été prélevés , mais tous se sont avérés négatifs c'est à dire que Mycobactérium Ulcérans n'a pu être mis en évidence dans ces prélèvements.

Tous les prélèvements réalisés dans la province du Nord-Ouest ont été détérioré à cause des mauvaises conditions de prélèvement . Il n'est donc pas exclu que l'ulcère de Buruli existe dans cette partie du pays d'autant que certains y provenant ont été retrouvés dans la province voisine du Sud-Ouest.

Il est fort probable que cette prévalence est sous estimée à cause des moyens limités , l'inaccessibilité dans certaines zones potentiellement endémiques et de l'état des routes en saison pluvieuse. A ces raisons s'ajoutent la mauvaise collaboration avec les tradi-praticiens, les interdictions imposées à certains malades par les guérisseurs et le scepticisme de la population envers la médecine moderne jugée d'inefficace.

Selon les données recueillies , la maladie semble être importante dans les villages traversés par le Nyong (de la province de l'Est au Centre).

Si les caractéristiques des foyers trouvés dans la province du centre sont similaires à ceux trouvés ailleurs, c'est à dire dans des zones marécageuses inondables il n'en va pas de même de ceux de la province de l'Adamaoua qui se développent sur un énorme bloc cristallin fracturé et soulevé situé à une altitude moyenne de 1100 m, avec un climat tropical humide (1500 mm de pluie par an) qui favorise la savane arborée. La température s'abaisse au fur et à mesure qu'on s'élève, et atteint dans certaines régions jusqu'à 20° .

Notre enquête a permis de déceler que 75% des lésions retrouvées dans notre étude sont au stade d'ulcère contre 92,8% dans l'enquête réalisée par Jürgen Noeske et collaborateurs dans leur enquête dans le bassin du Nyong en 2000 *. Les hôpitaux d'Ayos et Akonolinga sont devenus de grands centres de référence national où ont été orienté tous les cas dépistés lors de l'enquête .

Le délai moyen d'évolution de la lésions depuis l'apparition du nodule était de 10,3 mois et témoigne de la chronicité des lésions. En effet dans l'enquête sur les interprétations de l'ulcère de Buruli par les populations d'Ayos et d'Akonolinga, Dr Um B. et Deffo M. montrent que l'élément à mettre en cause qui rend peut-être compte de l'attentisme est l'absence de la douleur au début du développement de la maladie et selon une opinion très répandue dans l'ère culturelle Bantou, qui dit absence de douleur, dit absence de maladie. Toujours selon cette enquête , le bouton se déclare ; rien de bien grave se disent-ils ou mieux, cela passe.
En deuxième lieu, les démangeaisons apparaissent ; des idées nouvelles viennent se greffer à l'esprit, des conjectures jaillissent ; ça doit être une piqûre d'insecte –très souvent c'est le cas dans cet environnement forestier hostile à l'homme- Jusqu'ici, ce n'est rien de grave, inutile d'ameuter l'entourage familial.

Troisièmement, une plaie se profile à l'horizon ; c'est le stade terminal du processus dont on ne peut établir la durée avec exactitude. Ce stade très souvent est accueilli avec soulagement car la blessure va guérir au bout de quelques jour. Il n'est pas toujours nécessaire d'alerter qui que se soit, la redondance de ce phénomène pathologique dans ce milieu forestier hostile à l'homme a généré des formes de gestion intime et personnelle de la situation dans presque tous les groupes d'âges. Seulement, la plaie ne se referme pas pire encore, elle devient le siège d'un écoulement nauséabond ; la panique s'installe, forcé par les évènements, le malade extériorise son malaise pour une prise en charge qui commence dans la famille, s'étend à l'entourage avant de rejoindre en ultime recours le niveau de la prise en charge médicale. Sur le plan biomédical, on parlera déjà de notification tardive puis que ce n'est qu'au stade d'ulcère que la famille déclare la maladie à l'entourage. Ceci est vrai vu dans la perspective médicale mais sujet à caution une fois que l'on examine la situation du point de vue local.

En fait, les critères d'appréciation du temps ne convergent pas d'un côté comme de l'autre. le temps ne compte presque pas ; il ne s'agit pas de courir rapidement à l'hôpital mais plutôt, de laisser le monstre se dévisager totalement et même de s'apercevoir de sa nature obscure et son invincibilité sur le plan thérapeutique car chez les populations du Nyong et Mfoumou, comme dans bien d'autres univers de l'ère culturelle Bantou, le fait pour un individu à terme de faire preuve de polyvalence sociale c'est-à-dire, accumuler grâce à l'expérience et l'influence des anciens, un savoir polysémique (économie du groupe, santé, justice, technologie etc…) pour faire face au problème du quotidien. Chaque malade tout au long du déclenchement de la maladie, gère individuellement la situation, élabore successivement des hypothèses pour s'expliquer sur la nature du mal en faisant recours à sa propre expérience de vie et les connaissances accumulées tout au long de son parcours social. Inconsciemment, même les tous petits adhèrent à ce genre de comportement face à une maladie qui s'annonce sans provoquer à priori la douleur.

Il est évident que la faible couverture en infrastructure sanitaire de base, le manque de personnel qualifié pour la prise en charge de la maladie ainsi que le manque d'équipement et de matériel médical dans les formations sanitaire concourent à ce faible recours à la médecine moderne.

Les 115 cas non évolutifs observés présentent chacun un déficit . Il faut dire que dans l'ensemble, l'ulcère de Buruli est une importante source de déficits de mobilité entraînant très souvent des handicaps plus ou moins importants. Les causes des déficits de mobilité sont pour la plupart directement liées à une absence de rééducation appropriée. Elles sont le plus souvent le résultat d'un manque de mobilisation et/ou d'un positionnement incorrect tout au long du processus de guérison de la plaie et de sa cicatrisation.

Les rétractions et adhérences cutanées représentent la première cause des déficits de mobilité. La plupart des personnes touchées par l'ulcère présentaient, à différents degrés:

1. des rétractions cutanées
2. des adhérences touchant les tissus en profondeur
3. des rétractions capsulo-ligamentaires
4. des rétractions musculo-tendineuses.

L'ordre dans lequel sont mentionnées ces complications n'est pas fortuit : les deux dernières sont le plus souvent consécutives aux deux premières. Les rétractions cutanées peuvent même conduire à des déformations par luxation au niveau des

extrémités. Sur un échantillonnage de quelques dizaines de patients, près du 80% des troubles fonctionnels étaient secondaires aux problèmes cutanés. Sans rééducation, les résultats fonctionnels concernant les membres supérieurs semblent aussi médiocres après chirurgie qu'après une guérison spontanée ou un traitement par la médecine traditionnelle.

Selon Valérie Simonet dans son rapport exploratoire sur la rééducation à Ayos et Akonolinga, les patients dont les membres inférieurs ont été opérés précocement présentent moins de risque de perte de mobilité puisqu'une part de la rééducation s'effectue de façon spontanée avec la marche.

Dans le même rapport, elle précise que les membres supérieurs, par contre, présentent très souvent de graves atteintes fonctionnelles. L'explication de cet état de fait est à rechercher dans la complexité de leurs mouvements et de leur anatomie. De plus, il est plus aisé de se passer momentanément de l'un de ses bras, ce qui induit alors un manque de mobilisation qui favorise l'enraidissement.

Les patients avec des lésions dans le voisinage du coude, du poignet et/ou des doigts présentaient tous le même genre de déficit de mobilité. Il n'y avait pas de différence sensible entre ceux qui avaient été opérés, ceux qui avaient guéri spontanément ou ceux qui avaient fait appel à la médecine traditionnelle. Les problèmes de mobilité concernent le plus souvent ces mouvements : l'extension ou la flexion du coude, très fréquemment la supination, très fréquemment l'extension du poignet et plus rarement la flexion, presque systématiquement la flexion des articulations métacarpo-phalangiennes.

Enfin selon ce rapport , la lutte contre les rétractions cutanées demande une rééducation spécifique similaire à celle des grands brûlés.

9. CONCLUSION

La présente enquête a permis de montrer qu'il existe bien d'autres foyers confirmés d'ulcère de Buruli au Cameroun en dehors de la province du Centre . Après Ayos et Akonolinga , , le foyer de Mbalmayo dans la province du Centre mérite une attention particulière. La province du Centre est la plus endémique suivie de la province de l'Est et du Sud-Ouest .

La présence de la maladie dans les zones moins humides et non marécageuses comme l'Adamaoua zone pastorale par excellence, mérite une clarification à travers une étude environnementale de l'hôte.

Le facteur changement de l'environnement pourrait bien être mis en cause dans la présence de la maladie dans l'extrême -nord du pays où le climat est chaud et aride, mais on y retrouve un important barrage et une zone de grande culture de riz dans la région de Maga .

En tenant compte des données des centres de prise en charge d'Ayos et Akonolinga , la prévalence de la maladie est 930 cas d'ulcère de Buruli en 2004 au Cameroun.

L'enquête confirme toujours l'arrivée tardive des patients dans les formations sanitaires avec 75% des cas d'ulcère . En 2000 , Jürgen Noeske et collaborateurs trouvent dans leurs enquête réalisée dans le bassin du Nyong que 92,8% des patients sont diagnostiqués au stade d'ulcère . Une simple comparaison entre les deux enquête permet de constater une sensible amélioration de la sensibilisation des populations sur la maladie.

La connaissance traditionnelle de la maladie reste dominante et lié à un sort, cela impose une grande sensibilisation des populations à tous les niveaux.

Des efforts d'amélioration des infrastructures d'accueil en personnel qualifié et équipements et en consommables médicaux sont nécessaires pour faire naître la confiance des malades aux services de santé.

Du fait des limites de cette étude préliminaire, des études régionales plus ciblées méritent encore d'être menées afin de connaître la prévalence réelle de la maladie au Cameroun. Toutefois les données actuelles constituent une bonne base pour le développement du programme national de lutte contre cette maladie dans les nouveaux foyers.

10. ANNEXES

LISTE DES DISTRICTS, AIRES DE SANTE ET VILLAGES AYANT DES CAS CONFIRMES

10.1 Province de l'Adamaoua :

PROVINCE	DISTRICT	AIRE DE SANTE COUVERT	VILLAGES CONCERNES	CAS EVOLUTIFS	CAS NON EVOLUTIFS	TOUS CAS CONFONDUS
ADAMAOUA	BANKIM	BANDAM	Bandam , Nyakong	21	4	25
		NYAMBOYA	Nyamboya	7	0	7
		SOMIE	Mafoulou	5	1	6
	BANYO	BANYO	Sabongari	1	0	1
	MEIGANGA	MEIGANGA II	Meiganga	2	0	2
	TIGNERE	Libong	Libong	1	3	4
	NGAOUNDERE	BELEL	Bakari Bata-Sindere Laïde Kane	3	1	4
		Sassa Mberi	Sassa Mberi	2	0	2
**TOTAL**	**5 DISTRICTS**	**8 Aires de santé**	**10 villages**	**42**	**9**	**51**

10.2. Province du Centre

(hors mis les districts de santé d'Ayos et Akonolinga)

PROVINCE	DISTRICT	AIRE DE SANTE COUVERT	VILLAGES CONCERNES	CAS EVOLUTIFS	CAS NON EVOLUTIFS	TOUS CAS CONFONDUS
CENTRE	MBALMAYO	Mbalmayo	New town , quartier Haoussa	6	1	7
		Metet	Mbega , Mbedoumou II, Bizock Zalom, Yop, Olamdoé Ngoantet II, Nkol ekabli	187	12	199
		Ngomedzap	Adzap , Assie	13	2	15
	MFOU	Atega	Atega	4	0	4
		Dzeng	Fon, Edou Dzeng, Kamba , Ngouleman	15	7	22
		Komassi	Komassi , Bembe Ndzandouan	23	5	28
		Kamba	Kamba	17	8	25
		Ndangeung	Ndangeung III Ndangeung , Bembe , Nkolmeva'a	15	3	18
	NTUI	Nguilla	Nguilla	1	0	1
	ESEKA	Mom	Mom, Bakoukoue	8	3	11
		Eseka	Njock	3	0	3
	AWAE	Nkolessong	Elat	7	2	9
TOTAL	5 districts	12 aires de santé	31 villages	299	43	342

10.3. Province de l'Est :

PROVINCE	DISTRICT	AIRE DE SANTE COUVERT	VILLAGES CONCERNES	CAS EVOLUTIFS	CAS NON EVOLUTIFS	TOUS CAS CONFONDUS
**EST**	**ABONG MBANG**	**AKOK MAKA**	Landa , Mayos, Efoulan , Atok , Akok-Maka Kodjas,Dongo	176	53	229
**TOTAL**	1	1	7	176	53	229

10.4. Province de l'Extrême-Nord

PROVINCE	DISTRICT	AIRE DE SANTE COUVERT	VILLAGES CONCERNES	CAS EVOLUTIFS	CAS NON EVOLUTIFS	TOUS CAS CONFONDUS
**Extrême Nord**	**Maga**	**Pouss**	Pouss	1	1	2
**TOTAL**	1	1	1	1	1	2

10.5. Province du Sud

PROVINCE	DISTRICT	AIRE DE SANTE COUVERT	VILLAGES CONCERNES	CAS EVOLUTIFS	CAS NON EVOLUTIFS	TOUS CAS CONFONDUS
**SUD**	**EBOLOWA**	Ebolowa urbain	Angalé	1	0	1
	**SANGMELIMA**	Meyomessi	Meyomessi	3	0	3
	**Kribi**	Elog Batindi	Elogbatindi	1	0	1
TOTAL	**3 DISTRICTS**	**3 aires de santé**	**3 villages**	**5**	**0**	**5**

10.6. Province du Sud-Ouest :

PROVINCE	DISTRICT	AIRE DE SANTE COUVERT	VILLAGES CONCERNES	CAS EVOLUTIFS	CAS NON EVOLUTIFS	TOUS CAS CONFONDUS
SUD - OUEST	MUNDEMBA	MUNDEMBA	Meka	3	1	4
	KUMBA	KUMBA	Pulletin, Fiango	6	0	6
		MBONGUE	Mbongue	15	0	15
	EKONDO TITI	EKONDO TITI	Bekora, Njenku Besingi	19	5	24
	KUMBA	KUMBA	Mbonge , Mamfé (N-O) Fiango ,	21	3	24
	LIMBE	LIMBE	Ngeme	3	0	3
TOTAL	5 DISTRICTS	6 aires de santé	11 villages	67	9	76

11. BIBLIOGRAPHIE

Aguiar J, Stenou C (1997) Les ulcères de Buruli en zone rurale au Bénin : prise en charge de 635 cas. Med Trop 57 : 83-90

Amofah G, Asamoah S, Afram-Gyening C (1998) Effectiveness of excision of pré-ulcérative Buruli lesions in field situations in a rural district in Ghana. Trop Doct 28 (2): 81-83

Amofah G, Bonsu F, Tetteh C, Okrah J Asamoa K, Asiedu K, Addy J (2002) Buruli Ulcer in Ghana: Results of a National Case Search. Emerg Infect Dis 8 (2): 167-170

Anja Studer , Kaspar Wyss, Alphonse Um Boock (2004) L'ulcère de Buruli: identification des régions suspectes par une évaluation rapide de la situation au niveau national.

Asiedu K, Etuaful S (1998) Socioeconomic implication of Buruli ulcer in Ghana: a three-year review. Am J Trop Med Hyg 59 (6): 1015-1022

Asiedu K, Portaels F (2000) Introduction. Dans: Ulcère de Buruli : Infections à Mycobacterium ulcerans (eds. Asiedu K, Scherpbier R, Raviglione M) WHO/CDS/CPE/GBUI Geneva

Aujoulat I, Johnson C, Zinsou C, Guedenon A, Portaels F (2003) Psychosocial aspects of health seeking behaviours of patients with Buruli Ulcer in southern Benin. Trop Med Int Health 8 (8): 750-759

Barker D J P (1973) Epidemiology of Mycobacterium ulcerans infection. Trans R Soc Trop Med Hyg 67: 43-47

Bigelow J, Welling R, Sinnott R, Torres S, Evanson R (2002) Attitudes towards clinical and traditional treatment for the Buruli ulcer in the Ga district, Ghana (abstract). Ann African Med 1 (2)

Boisvert H (1977) L'ulcère cutanée à Mycobacterium ulcerans au Cameroun, II. Etude bactériologique. Bull Soc Path Exot 70 : 125-131

Darie H, Josse R, Kouakou H, Carbonnelle B, Asse h ? Kanga J M (1998) L'ulcère de Buruli : une mycobacteriose d'actualité. Bulletin de l'ALLF 3 : 5-10

Criaud J (1992) Géographie du Cameroun et de l'Afrique. Les classiques africains, Versailles Cedex

Darie H, Le Guyadec T, Touze J E (1993) Aspects épidémiologiques et cliniques de l'ulcère de Buruli en Côte d'Ivoire. A propos de 124 observations récentes. Bull Soc Path Ex 86 : 272-276

Debacker M, Aguiar J Steunou C, Zinsou C, Meyers WM, Guédénon A, Scott J T, Dramaix M, Portaels F (2004) Mycobacterium ulcerans Disease (Buruli Ulcer) in Rural Hospital, Southern Benin, 1997-2001. Emerg Infect Dis 10 (8) : 1391-1398)

Dobos K M, Quinn F D, Ashford D A, Horsburgh C R, King C H (1999) Emergence of a Unique Group of Necrotizing Mycobacterial Diseases. Emerg Infect Dis 5 (3): 367-378

Enquête de démographie et de santé 1998 , Bureau Central des Recensements et des Etudes de pipulation Ministère des Investissements Publics et de l'Aménagement du Territoire , DHS.

George K M, Chatterjee D, Gunawardana G, Welty D, Hayman J, Lee R, Small P L C (1999) Mycolactone: A Polyketide Toxin from Mycobacterium ulcerans required for Virulence. Science 283 (5403): 854

Hayman J (1991) Postulated Epidemiology of Mycobacterium ulcerans infection. Int J Epidemiology 20 (4): 1093-1098

Hayman J, Asiedu K (2000) Epidemiology. Dans : Ulcère de Buruli: Infections à Mycobacterium ulcerans (eds. Asiedu K, Scherpbier R, Raviglione M) WHO/CDS/CPE/GBUI Geneva

Hayman J, Mc Queen A (1985) The pathology of Mycobacterium ulcerans infection. Pathol 17: 594-600

Hutchinson M F, Nix H A, Mc Mahon J P, Ord KD (1996) Africa – a topographic and climate database (CD-Rom). The Australian National University, Canberra, ACT 0200, Australia

Idrisi 32 version I 32.22 (1987-2002), release two. GIS and Image Processing Software. Clarklabs Clark University Worcester, MA USA, www.clarklabs.com/www.idrisi.com

Johnson R C, Hougnihin, Guédenon A, Makoutode M, Ifebe D, Boko M, Portaels F(2004) Le traitement traditionnel de l'ulcère de Buruli au Bénin. Bull ALLF 14 : 37-38

Kanga J M, Kacou E D (2001) Aspects épidémiologiques de l'ulcère de Buruli en Côte d'Ivoire : résultats d'une enquête nationale. Bull Soc Pathol Exot 94 (1): 46-51

Mac Callum P, Tollhurst J C, Buckle G, Sisson H A (1948) A new mycobacterial infection in man. J Pathol Bacteriol 60: 93-122
MapInfo Professional Version 7.0, MapInfo Coorperation Troy, New York, USA

www.mapinfo.com

Marsollier L, Aubry J, Saint-André J-P, Robert R, Legras P, Manceau A-L, Bourdon S, Audrain C, Carbonnelle B (2003) Ecologie et mode de transmission de Mycocobacterium ulcerans. Path Bio 51 (8-9) : 490-495

Marston B J, Diallo M O, Horsburgh C R, Diomande I, Saki M Z, Kanga J M, Patrice G, Lipman H B, Ostroff S M, Good R C (1995) Emergence of Buruli ulcer disease in the Daloa region of Côte d'Ivoire. Am J Trop Med Hyg 52 (3) : 219-224

Meyers W M, Connor D H, McCullough B, Bourland J, Moris R, Proos L (1974) Distribution of Mycobacterium ulcerans infections in Zaïre, including the report of new foci. Ann Soc belge Méd trop 54 (3): 147-157

Meyers W M, Tignokpa N, Priuli G B, Portaels F (1996) Mycobacterium ulcerans infection (Buruli ulcer) : first reported patients in Togo. Br J Dermatol 134 (6): 1116-1121

Ministère de la Santé Publique (2004) Atelier de Finalisation du plan d'Action de Lutte contre l'ulcère de Buruli au Cameroun: Présentations et documents

NCCR North – South (2002/2003) Partenariats de recherche pour atténuer les syndromes du changement global

Ngos F (2002) Lutte contre l'ulcère de Buruli dans le Nyong et Mfoumou. Rapport annuel des activités

NOAA/ NASA Pathfinder Advanced Very High Resolution Radiometer (AVHRR) Land Project (University of Maryland) and the Distributed Active Center (Code 902.2) at Godded Space Flight Center, Greenbelt, MD 20771

Noeske J, Kuaban C, Rondini S, Sorlin P, Ciaffi L, Mbuabaw J Portaels F, Pluschke G (2004) Buruli Ulcer Disease in Cameroon rediscovered. Am J Trop Med Hyg 70 (5): 520-526

Oluwasanmi J O, Sulanke T F, Olurin E O, Itayemi S O, Alabi G O, Lucas A O (1976) Mycobacterium ulcerans (Buruli) skin ulceration in Nigeria. Am J Trop Med Hyg 25: 122-1228

OMS (2000) Ulcère de Buruli: Infection à Mycobacterium ulcerans (eds. Asiedu K, Scherpbier R, Raviglione M) WHO/CDS/CPE/GBUI Geneva

Pimsler M, Sponsler T A, Meyers W M (1988) Immunosuppressive properties of the soluble toxin from Mycobacterium ulcerans. J Infect Dis 157: 577-580

Portaels F, Aguiar J, Debacker M, Steunou C, Guedenon A, Meyers W M (2002) Prophylactic effect of mycobacterium ulcerans disease (Buruli Ulcer). Clin Diagn Lab Immunol 9 (6): 1389-1391

Portaels F, Guimaraes-Pers A, Fonteyne P-A, Meyers W M (1999) Insects in the transmission of Mycobacterium ulcerans infection. Lancet 353: 986

Ravisse P (1977) L'ulcère cutané à Mycobacterium ulcerans au Cameroun, I. Etude clinique. Bull Soc Path Exot 70 : 109-124

Ravisse P, Roques M C, Le Bourthe F, Tchuembou C J, Menard J C (1975) Une Affection méconnue au Cameroun, l'ulcère à mycobactérie. Med Trop 35 (6) : 471-474

Ross B C, Johnson PDR, oppedisanoF, Marino L, Sievers A, Stinear T, Hayman J A, Veitch M G K, Robins-Browne R M (1997) Detection of Mycobacterium ulcerans in

Smith P G, Revill W D, Lukwago E, Rykunshin Y P (1977) the protective effect of BCG against Mycobacterium ulcerans disease: a controlled trial in an endemic area of Uganda. Trans R Soc Trop Med Hyg 70 (5-6): 449-457

Stienstra Y, van der Graaf W T A, Asamoa K, van der Werf T (2002) Beliefs and attitudes towards Buruli Ulcer in Ghana. Am J Trop Med Hyg 67 (2) : 207-213

Uganda Buruli Group (1971) Epidemiology of Mycobacterium ulcerans infection (Buruli Ulcer) at Kinyara, Uganda. Trans R Soc Trop Med Hyg 65 (6): 763-775

Um Boock A, Deffo M (2004) Interprétation de l'ulcère de Buruli par les populations d'Ayos et d'Akonolinga dans la province du Centre au Cameroun

Vallléry Simonet : Rapport d'évaluation des besoins en rééducation sur l'ulcère de Buruli à Ayos et Akonolinga . décembre 2003

Van der Graaf W T, Scherpbier R, ed. (1999) Buruli ulcer (Mycobacterium ulcerans infection) Report from the International Congress in Yamoussoukro, Ivory Coast 1998. WHO Document (WHO/TB/98.252)

Van der Werf T S, Stinear T, Stienstra Y, van der Graaf W T A, Small P (2003) Mycolactones and Mycobacterium ulcerans disease. Lancet 362: 1062-1064

Van der Werf T S, van der Graaf W T A, Tappero J W, Asiedu K (1999) Mycobacterium ulcerans infection. Lancet 354: 1013-1018

Veitch M G, Johnson P D, Flood P E, Leslie D E, Street A C, Hayman J A (1997) A large localized outbreak of Mycobacterium ulcerans infection on a temperate southern Australian island. Epidemiol Infect 119 (3): 313-318

WHO/CDS/CPE/GBUI/2004.4 Diagnostic de l'infection à Mycobacterium ulcerans (ulcère de Buruli)

WHO/OMS (2004) Ulcère de Buruli (Infection à Mycobacterium ulcerans : atlas des cas déclarés dans le monde). Relève Epidémiologique hebdomadaire 20 : 194-199
